# MicroRNA‐375‐3p Targets Fatty Acid Synthase and Relish to Regulate Energy Allocation During Pupal Metamorphosis and Starvation

**DOI:** 10.1002/advs.202513486

**Published:** 2026-02-12

**Authors:** Peng Chen, Meiqi Cheng, Jianhui Wang, Jing Tang, Xiaoqiao Huang, Yusi Li, Huiling Zhou, Ling Zhang, Yi Dong, Chengjun Li, Bin Li

**Affiliations:** ^1^ College of Life Sciences Nanjing Normal University Nanjing China; ^2^ Anhui Finance & Trade Vocational College Hefei Anhui China

**Keywords:** energy reallocation, insect, metabolic adaptation, miR‐375‐3p

## Abstract

Energy reallocation is critical for organismal survival under developmental transitions and nutrient restriction. However, whether developmentally and nutritionally induced energy deficiency are governed by convergent regulatory mechanisms remains poorly understood. Here, we identify miR‐375‐3p as a central regulator that coordinates energy use during both pupal metamorphosis and starvation using the *Tribolium castaneum*. Upregulated by endocrine cues including 20‐hydroxyecdysone and insulin, miR‐375‐3p redirects energy by suppressing de novo lipogenesis through fatty acid synthase (*FASN*) and enhancing lipolysis via *Relish* inhibition. This regulatory shift mobilizes stored reserves while suppressing energetically costly immune and oxidative responses, thereby prioritizing energy supply to the central nervous system. Under fed conditions, miR‐375‐3p levels decline, restoring energy distribution across all tissues and enabling immune competence. These findings reveal a conserved miRNA‐centered mechanism that mediates metabolic adaptation to energy scarcity and provide new insights into miRNA‐driven control of energy balance.

## Graphical abstract

During pupal metamorphosis and starvation, elevated 20‐hydroxyecdysone (20E) and suppressed insulin trigger Forkhead box O (FOXO) nuclear translocation, enhancing miR‐375‐3p expression. This downregulates *fatty acid synthase* (*FASN*) and *Relish*, promoting lipid breakdown for energy while prioritizing antioxidant responses over immune functions to support survival under energy deficiency.

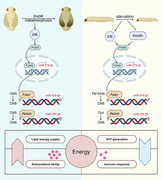

## 1 Introduction

Energy metabolism is fundamental to sustaining physiological functions [1], and disruptions in energy balance can have profound effects on cellular and systemic homeostasis [2]. Organisms have evolved intricate mechanisms to sense and respond to energy fluctuations [3, 4, 5], ensuring that even under adverse conditions, essential processes such as growth, development, repair, and reproduction are sustained through efficient energy allocation [6]. Understanding the regulatory networks underlying these adaptive responses is not only essential for advancing basic biological research, but also crucial for addressing metabolic diseases associated with energy imbalances.

In insects, pupal metamorphosis and starvation are two distinct yet informative models for studying energy deficiency. Pupal metamorphosis represents a natural state of energy isolation, during which organisms undergo extensive physiological reorganization to complete developmental transitions [7, 8]. In contrast, starvation is an externally induced condition of energy scarcity that forces organisms to prioritize essential functions for survival [9, 10]. Despite their different triggers, both conditions demand precise reallocation of energy resources, raising the question of whether convergent regulatory mechanisms govern these adaptive responses.

Lipid metabolism plays a pivotal role in energy allocation [11]. Triacylglycerol (TAG) is a key component of lipid metabolism and the primary energy source from dietary lipids [12, 13]. TAG plays a crucial role in mediating vital physiological processes, including molting [14, 15], eclosion [16, 17, 18], immune responses [19], and antioxidation [20, 21]. Additionally, TAG levels are tightly linked to energy availability and deficiency, including starvation and pupal metamorphosis [22, 23, 24]. For example, during the pupal stage, lipid reserves function as the primary fuel source and as precursors for membrane lipid synthesis during metamorphosis [25], and during starvation, stored lipids in the midgut are depleted [8]. Thus, alterations in lipid metabolism play a critical role in both forms of energy deficiency. However, whether these changes are governed by a shared regulatory mechanism remains unclear.

miRNAs are small, non‐coding RNA molecules that modulate gene expression post‐transcriptionally, playing a crucial role in fine‐tuning cellular processes [26, 27]. Recent studies have uncovered that miRNAs are key players in metabolic adaptation, including lipid metabolism, energy expenditure, and oxidative stress management during both developmental transitions and environmental stressors. For example, in *Bombyx mori*, miR‐281 represses EcR‐B, an important regulator of energy homeostasis during metamorphosis, indicating that miRNAs coordinate metabolic shifts in response to developmental cues [28]. In *Bactrocera dorsalis*, miR‐275/305 cluster regulates insulin signaling, suggesting that miRNAs can modulate insulin response pathways to control metabolic processes like lipogenesis and gluconeogenesis [29]. Additionally, miRNAs such as miR‐20b are involved in metabolic switches between lipids and carbohydrates, with miR‐20b modulating fatty acid synthesis in chicks during delayed feeding, in interaction with FOXO3, a key transcription factor in metabolic stress response [30]. Moreover, miR‐155 has been shown to regulate FOXO3, influencing metabolic stress responses in hypertension [31]. These studies underscore the importance of miRNAs as regulators of metabolic networks that balance lipid storage, energy production, and cellular survival during stress.

Here, we selected *T. castaneum*, a typical holometabolous insect with well‐defined developmental stages and conserved lipid metabolism pathways, as an ideal model for studying miRNA‐mediated regulation of energy metabolism and metamorphosis. Our study reveals that miR‐375‐3p is highly expressed during pupal metamorphosis and starvation in *T. castaneum*. miR‐375‐3p modulates lipid metabolism by targeting *FASN* and *Relish*, key components of lipid synthesis and immune function. Moreover, miR‐375‐3p enhances antioxidant capacity through the upregulation of catalase (CAT), which helps mitigate oxidative damage during energy deprivation. FOXO, a well‐known transcription factor involved in metabolic stress responses, regulates miR‐375‐3p expression by binding to its promoter region. This FOXO‐miR‐375‐3p axis links transcriptional control with post‐transcriptional regulation, providing a robust mechanism that ensures proper energy allocation under conditions of metabolic stress. Together, these findings highlight how miRNAs, especially miR‐375‐3p, serve as central modulators of energy metabolism, coordinating lipid homeostasis, immune responses, and oxidative stress management during developmental and environmental challenges.

## 2 Results

### 2.1 miR‐375‐3p is a Crucial Regulator of Pupal Metamorphosis in *Tribolium Castaneum*

**FIGURE 1:**
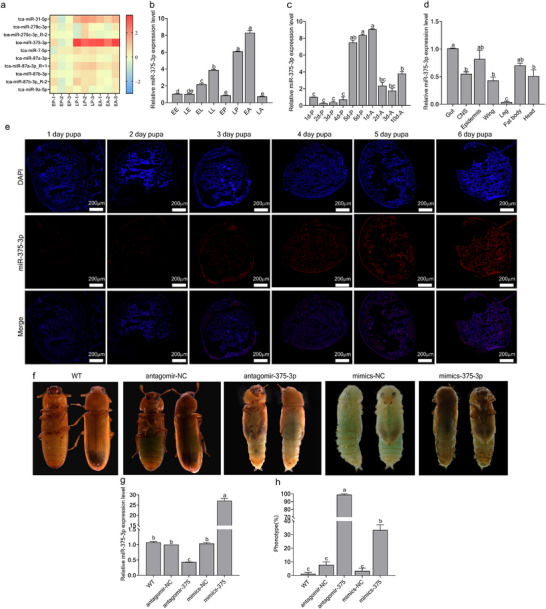
miR‐375‐3p is a crucial miRNA in the eclosion of *T.castaneum*. (a) Significant differentially expressed miRNAs screened by miRNA‐seq from EP, LP, and EA. Color scale indicates the number of standard deviations from the mean of all lines. (b) The expression level of miR‐375‐3p in different stages of *T. castaneum* (whole body). The developmental stage samples included 1‐day‐old eggs (EE, ∼50 mg), 3‐day‐old eggs (LE, ∼50 mg), 1‐day‐old larvae (EL, ∼50 mg), 20‐day‐old larvae (LL, 3 individuals), 1‐day‐old pupae (EP, 3 individuals), 5‐day‐old pupae (LP, 3 individuals), 1‐day‐old adults (EA, 3 individuals), and 10‐day‐old adults (LA, 3 individuals). (c) The expression level of miR‐375‐3p from pupal stage to adult stage in *T. castaneum*(whole body).1d‐P to 6d‐P: 1‐ to 6‐day‐old pupae. 1d‐A to 3d‐A: 1‐ to 3‐day‐old adults. 10d‐A: 10‐day‐old adults. (d) The expression pattern of miR‐375‐3p in different tissues of LP. Tissues include: gut, central nervous system (CNS), epidermis, wing, leg, fat body and head. (e) The in situ hybridization results from 1‐day‐old to 6‐day‐old pupae indicate a gradual increase in miR‐375‐3p expression levels, the red fluorescence signal represents miR‐375‐3p, and the blue fluorescence signal represents DAPI. (f) Phenotypes and morphological differences after injection of antagomir‐NC, antagomir‐375‐3p, mimics‐NC and mimics‐375‐3p. (g) The expression level of miR‐375‐3p after injection of antagomir‐NC, antagomir‐375‐3p, mimics‐NC and mimics‐375‐3p. (h) The phenotype rate after injection of antagomir‐NC, antagomir‐375‐3p, mimics‐NC and mimics‐375‐3p (The error bar represents the mean ± SEM of three biological replicates. The comparison between multiple sets of data was analyzed by one‐way ANOVA followed with Tukey's test).

Our previous small RNA transcriptome sequencing identified 550 differentially expressed miRNAs across three developmental stages of *T. castaneum* [32]. Notably, miR‐375‐3p exhibited the most significant change, with low expression at the 1‐d pupae (early pupae, EP) stage but was significantly upregulated from 6‐d pupae (late pupae, LP) to 1‐d adults (early adults, EA) (Figure 1a). qPCR analysis confirmed that miR‐375‐3p was minimally expressed in EP but abundantly expressed in LP and EA, with prominent localization in the fat body, epidermis, and gut of LP (Figure 1b–d). In situ hybridization revealed a progressive increase in miR‐375‐3p expression from 1‐day‐old to 6‐day‐old pupae (Figure 1e). To explore its functional role, we injected pupae with mimics‐375‐3p and antagomir‐375‐3p, respectively. Overexpression of miR‐375‐3p by mimics‐375‐3p caused 33.3% of pupae failed to complete eclosion, while knocking down it by antagomir‐375‐3p resulted in a failure of 98.9% of pupae in initiating eclosion (Figure 1f–h). Collectively, these results demonstrated that miR‐375‐3p is a crucial regulator of pupal metamorphosis in *T. castaneum*.

### 2.2 miR‐375‐3p Plays a Key Role in Starvation Response in *T. castaneum*

A notable feature of pupal metamorphosis is the absence of external energy intake, with its regulation being mediated by 20‐hydroxyecdysone (20E). For example, in *Helicoverpa armigera*, starvation represents an externally induced nutrient‐deficient state that also involves 20E‐mediated regulation, leading to cellular responses such as autophagy and apoptosis [33]. The parallel between these two energy deficient conditions raises the question of whether miR‐375‐3p plays a conserved role in energy regulation across different energy‐deficient states.

**FIGURE 2:**
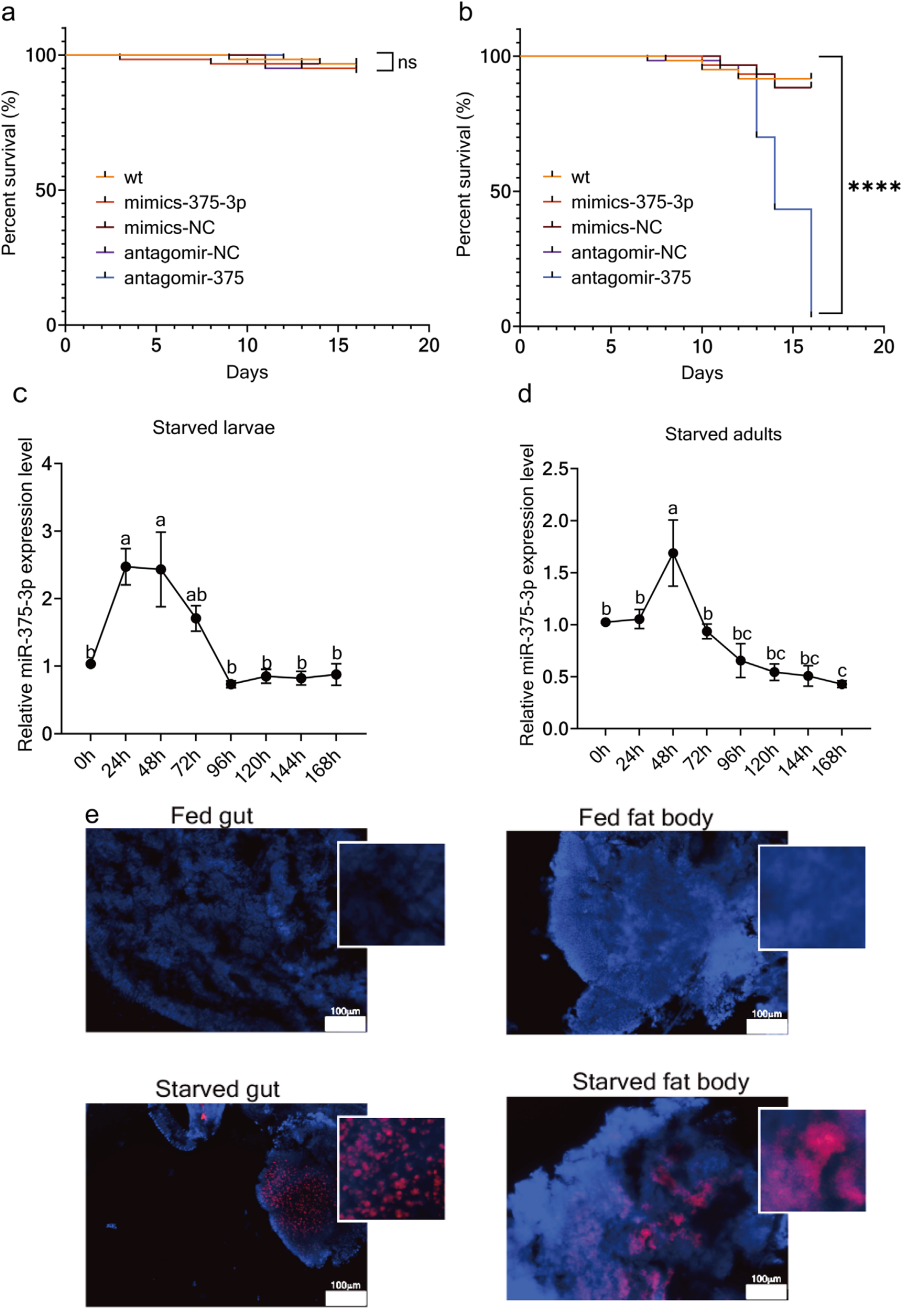
High expression of miR‐375‐3p is essential for maintaining survival during starvation. (a,b) Survival of WT, antagomir‐NC, antagomir‐375‐3p, mimics‐NC and mimics‐375‐3p with feeding or starvation treatment. All survival curves were plotted and analyzed by Log‐rank (Mantel‐Cox) test using GraphPad Prism 9. 90 individuals were used for all groups. n.s., not significant; ^****^ *p* < 0.0001. (c) The expression level of miR‐375‐3p in larvae under starvation treatment in *T. castaneum* (whole body). (d) The expression level of miR‐375‐3p in adults under starvation treatment in *T. castaneum* (whole body). (e) In situ hybridization showed the expression of miR‐375‐3p higher in starved gut and fat body compared to feeding treatment, the red fluorescence signal represents miR‐375‐3p, and the blue fluorescence signal represents dapi. The significant differences among different treatments are indicated by lowercase letters above each bar (one‐way ANOVA followed with Tukey's test, different letters indicate significant differences within treatments).

To address this, we subjected 15‐day‐old larvae, a high metabolic demand stage, to starvation treatment and following injection with mimics‐NC, mimics‐375‐3p, antagomir‐NC, or antagomir‐375‐3p. The starved group with miR‐375‐3p knockdown exhibited nearly 100% mortality within 15 days, whereas fed groups showed no significant differences in survival (Figure 2a,b). These results highlight miR‐375‐3p as essential for survival under starvation. qPCR analysis revealed a significant upregulation of miR‐375‐3p in both larvae and adults after 48 h of starvation (Figure 2c,d), and in situ hybridization further confirmed increased miR‐375‐3p expression in the gut and fat body that are crucial for energy allocation under starvation conditions (Figure 2e). These findings indicated miR‐375‐3p as a key regulator of the starvation response by facilitating energy allocation and enhancing survival under energy deficient conditions.

### 2.3 miR‐375‐3p Directly Targets *FASN* and *Relish*

**FIGURE 3:**
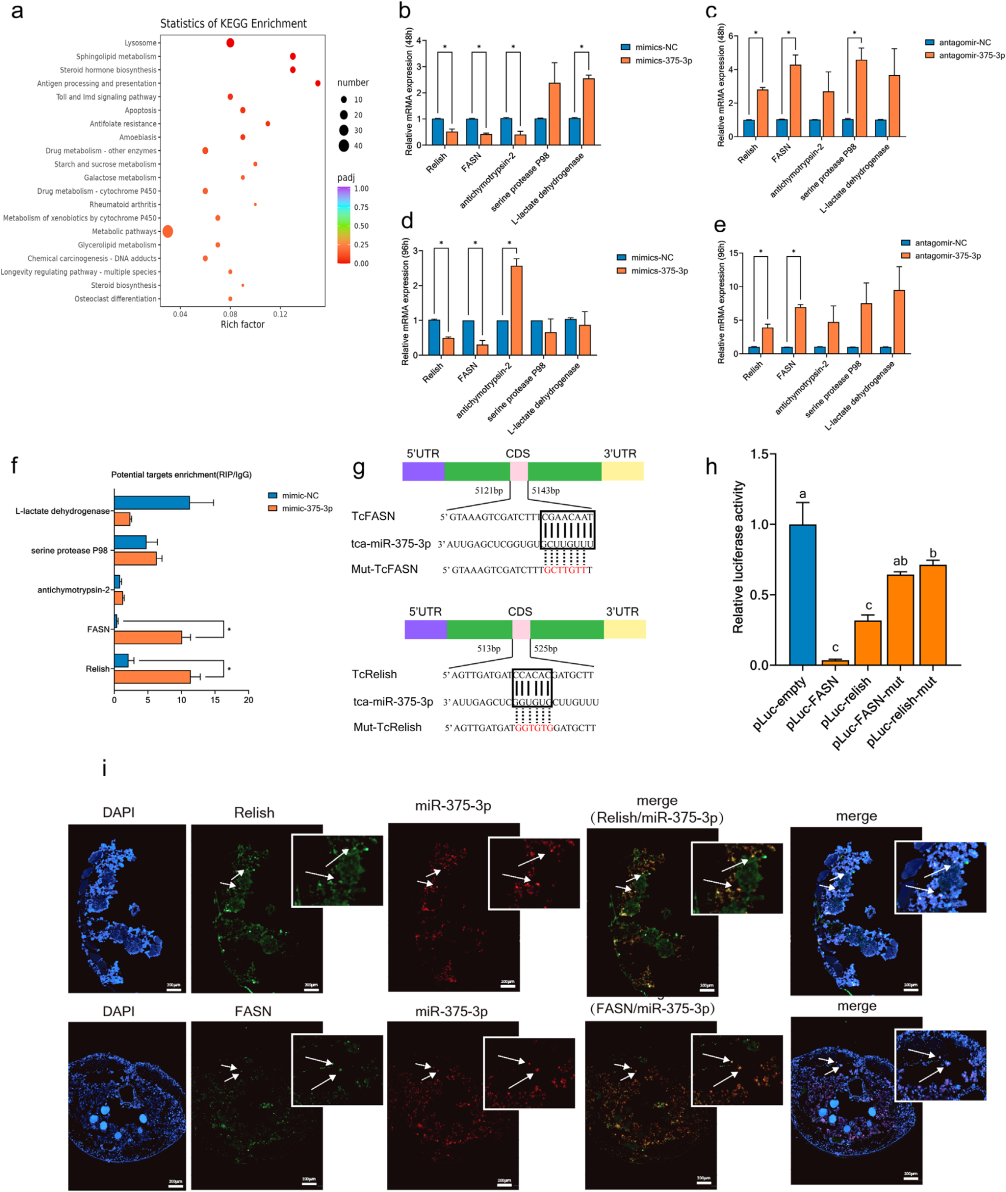
miR‐375‐3p targets *FASN* and *Relish* transcript in vivo. (a) Top 20 pathways from the KEGG enrichment analysis of predicted candidate miR‐375‐3p targeting DEGs identified through genome‐wide RNA‐Seq between antagomir‐NC and antagomir‐375‐3p beetles. (b–e) Transcript levels of candidate target genes at 48 and 96‐h after overexpression or knockdown of miR‐375‐3p. (f) Verification of target genes by RIP in beetles injected with mimics‐375‐3p or mimics‐NC for 48 h. Normal mouse IgG was used as a negative control. (g) The putative tca‐miR‐375‐3p binding sites in *FASN* and *Relish* were predicted by miRanda and RNAhybrid. (h) Luciferase reporter assays in S2 cells cotransfected with miR‐375‐3p mimics and pLuc vectors containing WT or mutant sequences of *FASN* and *Relish*. (i) In situ hybridization and immunofluorescence reveal the co‐localization of miR‐375‐3p, FASN, and Relish, with blue representing the nucleus, green representing the target gene, red representing miR‐375‐3p, and arrows pointing to co localization points. The results of the qPCR, RIP and luciferase activity analyses are presented as mean ± SEM (*n* = 3). The significant differences among different treatments are indicated by lowercase letters above each bar (one‐way ANOVA followed with Tukey's test, *p* < 0.05 for a, b, and c, ^*^ *p* < 0.05).

To identify the target genes related to the biological effects of miR‐375‐3p, we only considered the binding and expression relationship between miR‐375‐3p and differential expression genes (DEGs) identified from transcriptomic analysis of antagomir‐NC and antagomir‐375‐3p beetles. A total of 157 candidate target genes were found among the DEGs between antagomir‐NC and antagomir‐375‐3p beetles (Dataset S1). Furthermore, Kyoto Encyclopedia of Genes and Genomes (KEGG) enrichment analysis showed that 27 candidate target genes were potentially bound to miR‐375‐3p (Figure 3a; Figure S2a,b and Dataset S1 and S2).

To further screen, we employed the miRanda algorithm (http://www.microrna.org/microrna/home.do) and the RNAhybrid program to predict the potential target genes of miR‐375‐3p. Among the 27 candidates, *serine protease P98* (LOC662682), *fatty acid synthase* (*FASN*, LOC103314884), *antichymotrypsin‐2* (LOC661409), *Relish* (LOC659499) and *L‐lactate dehydrogenase* (LOC656589) were predicted to bind with miR‐375‐3p (alignment score > 115, *p* value <0.05, Dataset S3) (Figure S2c). The qPCR analysis revealed that only *FASN* and *Relish* expression were increased or reduced after 48 and 96 h post injection with antagomir‐375‐3p or mimics‐375‐3p, suggesting that *FASN* and *Relish* were negatively regulated by miR‐375‐3p (Figure 3b–e). RNA immunoprecipitation (RIP) assay found that *FASN* and *Relish* were significantly enriched in the Ago1‐immunoprecipitated RNAs from beetles treated with mimics‐375‐5p compared with those from beetles treated with mimics‐NC (Figure 3f). We discovered potential miR‐375‐3p binding sites in the coding sequences (CDS) of FASN and Relish (Figure 3g). Dual‐luciferase reporter assays indicated that co‐transfection of mimic‐miR‐375‐3p with the CDS regions of FASN or Relish containing the predicted binding sites significantly reduced luciferase activity, and the reporter activity recovered when the construct containing the mutated FASN or Relish sequences (Figure 3h). Both in situ hybridization and immunofluorescence assays revealed the co‐localization of miR‐375‐3p with FASN and Relish in late pupal tissues (Figure 3i). These results conclusively demonstrated that *FASN* and *Relish* are direct targets of miR‐375‐3p in *T. castaneum*.

### 2.4 miR‐375‐3p Negatively Regulates *FASN* and *Relish* to Mediate the Lipid Metabolism

**FIGURE 4:**
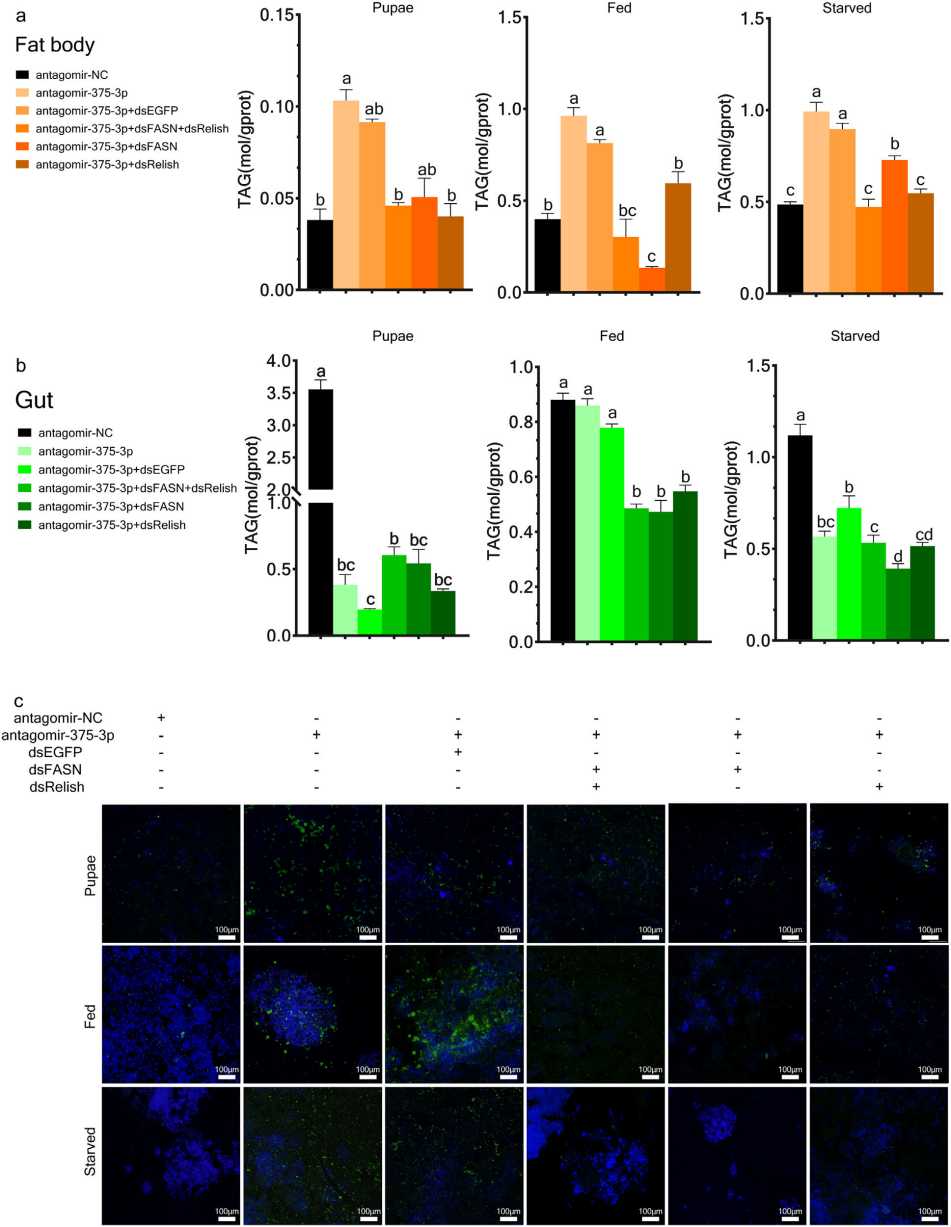
miR‐375‐3p negatively regulates *FASN* and *Relish*, inhibits fatty acid production and promotes lipid breakdown. (a) Determination of triglyceride content in the fat body during feeding larvae, starvation larvae and pupal stages. (b) Determination of triglyceride content in the gut during feeding larvae, starvation larvae and pupal stages. (c) BODIPY staining of lipid droplets (green) in the fat body during feeding larvae, starvation larvae and pupal stages. The significant differences among different treatments are indicated by lowercase letters above each bar (one‐way ANOVA followed with Tukey's test, *p* < 0.05 for a, b, and c).

In *Drosophila*, FASN plays a central role in de novo lipogenesis [34], while Relish regulates TAG lipolysis by suppressing the expression of *Brummer* (*Bmm*) [35]. Based on these clues, we hypothesized that miR‐375‐3p likely regulates the lipid metabolism by targeting *FASN* and *Relish*. We examined the TAG content in the fat body and gut of larvae and pupae subjected to different treatments. In the fat body, treatment with antagomir‐375‐3p and antagomir‐375‐3p+*dsEGFP* significantly increased TAG levels compared to the antagomir‐NC group, and knockdown of *FASN* and *Relish* reversed the increase in TAG levels (Figure 4a). In the gut, all treatments in the pupal and starved groups largely reduced TAG levels, while only the treatments with antagomir‐375‐3p + *dsFASN* + *dsRelish*, antagomir‐375‐3p+*dsFASN*, and antagomir‐375‐3p + *dsRelish* remarkably decreased TAG levels in fed larvae (Figure 4b). Interestingly, despite antagomir‐375‐3p and antagomir‐375‐3p + dsEGFP treatments, TAG levels in the pupal stage still decreased significantly. This could be attributed to impaired intestinal lipid absorption. Insects hydrolyze dietary triglycerides into free fatty acids (FAs) and glycerol in the midgut, with these FAs then being re‐esterified into TAGs in the endoplasmic reticulum of midgut epithelial cells. Our observations suggest that miR‐375‐3p knockdown impairs this process, limiting the re‐esterification of fatty acids and reducing intestinal TAG synthesis. Consequently, significant reductions in TAG levels were observed in pupae under starvation, but not in the feeding group, where sufficient external nutrient intake compensated for the impaired absorption.

Accumulation of food residues was observed in the midgut of insects treated with antagomir‐375‐3p. We speculate that this may result from impaired absorption and processing of triacylglycerol (TAG) in the midgut due to the suppression of miR‐375‐3p, particularly under conditions of energy deficiency, as TAG hydrolysis normally generates free fatty acids (FFAs) for energy supply [36]. However, under feeding conditions, this effect was less pronounced, likely due to the masking of the impairment by exogenous nutrient supplementation (Figure S3). Lipid droplet staining further confirmed that miR‐375‐3p inhibition increased lipid droplet accumulation, while simultaneous knockdown of *FASN* or *Relish* reversed this effect (Figure 4c).

Under feeding conditions, knockdown of *FASN* and *Relish* resulted in a significant decrease in TAG levels, accompanied by the reduction in body length, while the weight gain rate remained stable (Figure S4b–f). Lipid droplet staining revealed a significant reduction in droplet size under both feeding and starvation conditions, indicating impaired lipid storage capacity (Figure S4g). Taken together, these results demonstrated that miR‐375‐3p mediates lipid metabolism by regulating its direct targets, *FASN* and *Relish*, particularly under a state of energy deficiency.

### 2.5 miR‐375‐3p Maintains Low‐level of Immune Response and Oxidative Stress During Pupal Stage or Under Starvation Conditions

Given the importance of Relish as a key immune transcription factor, we further investigated whether miR‐375‐3p influences the immune response. To explore this, we infected *T. castaneum* with *Escherichia coli* and *Staphylococcus aureus*. The experiment was conducted during the pupal stage, feeding and starvation conditions. The results showed that silence of miR‐375‐3p during the pupal stage and starvation conditions significantly decreased colony counts (Figure S5a,b), while the expression of *Relish* and related antimicrobial peptides was significantly upregulated (Figure S5c,d). In the fed group, overexpression of miR‐375‐3p led to a significant increase in colony counts, while the expression of *Relish* and related antimicrobial peptides was significantly downregulated (Figure S5).

Transcriptomic data indicated a significant decrease in catalase (*CAT*) expression under miR‐375‐3p regulation, suggesting its involvement in oxidative stress modulation (data not shown). In the starved larvae and pupae, miR‐375‐3p knockdown significantly reduced CAT activity and total antioxidant capacity (T‐AOC). In contrast, in the fed larvae, overexpression of miR‐375‐3p resulted in increased CAT and T‐AOC activity (Figure S5e,f). Reactive oxygen species (ROS) levels, assessed by H2DCFH staining, displayed an opposite trend: miR‐375‐3p knockdown largely increased ROS in the pupal and starved groups, while overexpression of it reduced ROS levels in the fed group (Figure S5g). These results suggested that miR‐375‐3p maintained the low‐level of immune response and oxidative stress under the two different energy deficiency exists of pupal stage and starvation conditions.

### 2.6 miR‐375‐3p Preferentially Supplied ATP in central Nervous System During Pupal Stage or Under Starvation Conditions

The qPCR results indicated high expression of *FASN* and *Relish* in the central nervous system (CNS) during starvation and pupal metamorphosis (Figure S6). It was discovered that antagomir‐375‐3p treatment remarkably elevated the overall ATP content in fed larvae but not in the pupal stage and starved larvae (Figure S7a–c). To examine energy allocation, we assessed ATP distribution in various tissues in 6‐day‐old pupae as well as 15‐day larvae 48 h post feeding and starvation treatments. In pupae and starved larvae, knockdown of miR‐375‐3p increased the ATP content in the CNS but decreased it in the fat body and gut (Figure S7d,f). In the fed larvae, miR‐375‐3p knockdown increased the ATP content in the CNS, gut, and fat body (Figure S7e). This redistribution may reflect a physiological adaptation in which miR‐375‐3p functions as a central regulator of energy allocation, coordinating the balance between lipid metabolism and ATP production across tissues, particularly by regulating energy metabolism in the central nervous system and intestine during energy‐deficient conditions such as starvation and pupal transformation.

### 2.7 FOXO Directly Binds to the miR‐375‐3p Promoter to Regulates Immune Responses and Oxidative Stress During Pupal Stage and Under Starvation Conditions

Unlike traditional mRNA genes, the promoter regions of miRNA are widely unknown in *T. castaneum*. To overcome this limitation, we adapted a pri‐miRNA enrichment method, RNA interference (RNAi) used to knock down the *T. castaneum drosha* transcript to enriching pri‐miRNAs [37, 38]^)^. Next, the total RNA of *dsDrosha* beetles was used for 5’‐rapid amplification of cDNA ends (5’‐RACE) to identify novel pri‐miRNAs and their potential transcription start sites (TSS) [39]. qPCR analysis indicated that the *Drosha* transcripts were reduced by 60% after injecting its dsRNAs (Figure S8a). We identified the TSS of miR‐375 at a guanine (G) located 733 bp upstream of pre‐miR‐375‐3p. A schematic representation of this genomic region is provided in the (Figure S8b).

**FIGURE 5:**
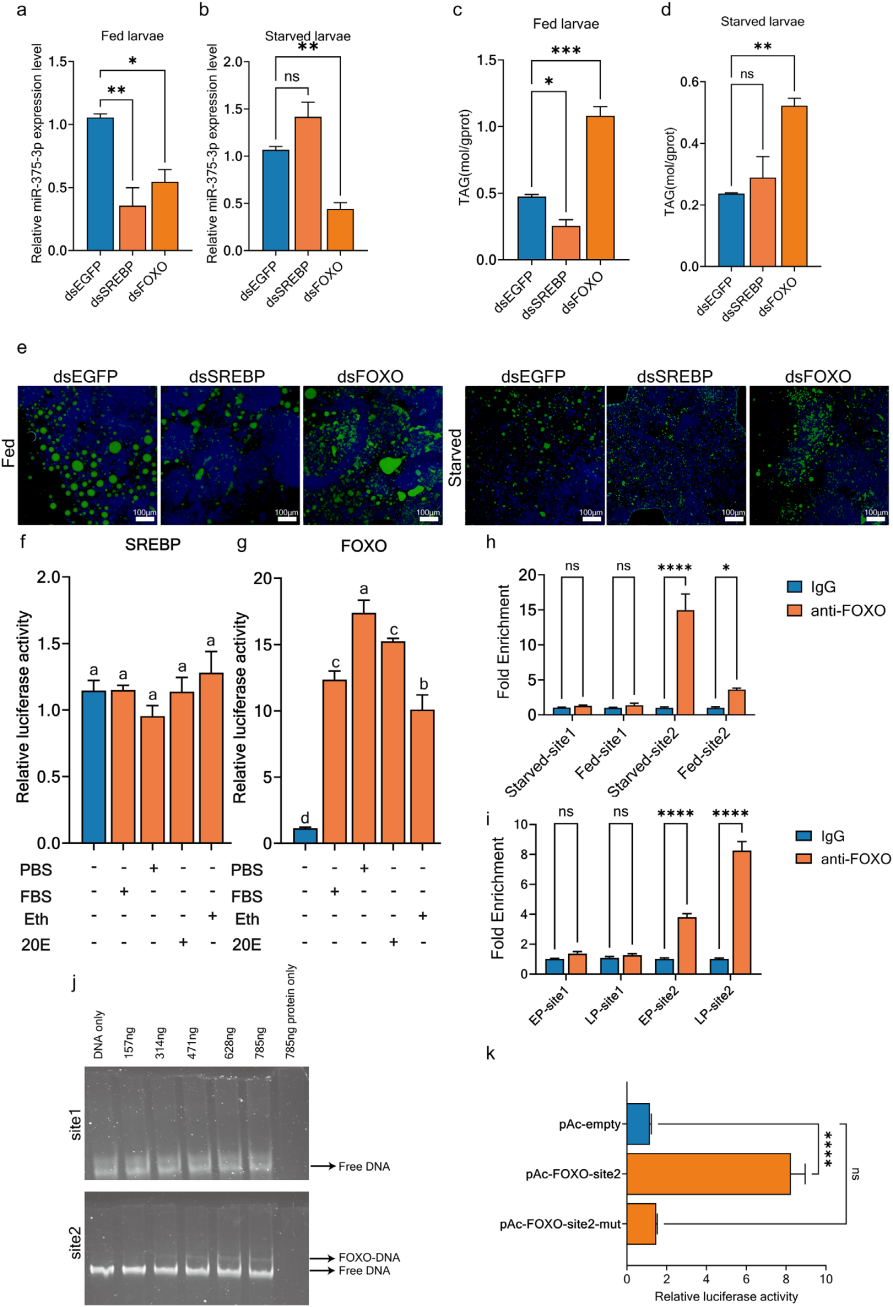
FOXO directly targets the promoter region of miR‐375‐3p. (a,b) miR‐375‐3p expression levels after *SREBP* and *FOXO* knockdown under starvation and feeding treatments. (c,d) Determination of and triglyceride content during feed larvae and starved larvae. (e) Lipid droplet staining of beetles during feed larvae and starved larvae. (f,g) Luciferase assays in S2 cells transfected with the pGL3 Basic reporter plasmid carrying the miR‐375‐3p promoter and the expression vectors for SREBP or FOXO. Treatments with empty pAc5.1 expression vector served as controls. A Renilla luciferase reporter construct was cotransfected in each well as a reference. Transfected cells were exposed to 20E (10^−6^ m) dissolved in ethanol (Eth) or Eth alone and supplemented with 10% fetal bovine serum (FCS) or PBS. (h,i) ChIP‐qPCR was performed to detect the fold change in FOXO binding on the promoter of miR‐375‐3p, experiments were conducted to extract chromatin from the starvation group, feeding group, early pupae, and late pupae separately. (j) EMSA using miR‐375‐3p promoter fragment and recombinant TcFoxO protein. (k) A dual‐luciferase assay was performed to evaluate the binding activity of pAc‐FOXO, pAc‐FOXO mutant, and pGL‐miR‐375‐3p‐promoter. (Error bars represent SEM. one‐way ANOVA followed with Tukey's test. ^****^ *p* < 0.0001, ^***^ *p* < 0.001, ^**^ *p* < 0.01 ^*^ *p* < 0.05 and ns means no significance).

The 1.5 kb sequence upstream of its promoter was analyzed using the Jaspar database (https://jaspar.elixir.no/) to predict potential binding sites (Dataset S4). And the top nine highest‐scoring sites were selected for further validation (Dataset S5). At the 3day pupal stage, we knocked down each of the nine transcription factors and assessed knockdown efficiency and miR‐375 expression 48 h later. All nine knockdowns were successful, but only *SREBP* or *FOXO* knockdown exhibited a significant reduction in miR‐375‐3p expression during the pupal stage (Figure S8c,d). Under feeding conditions, knockdown of *SREBP* or *FOXO* decreased the expression of miR‐375‐3p, however, in starved larvae, only *FOXO* knockdown reduced its expression (Figure 5a,b). Similar to the miR‐375‐3p knockdown, silence of *FOXO* resulted in a significant increase in TAG content and lipid droplet size group, with (Figure 5c–e).

We cloned the 5′ upstream regulatory region (−1499 to +23) of miR‐375‐3p into the pGL3 Basic luciferase reporter vector. The coding sequences (CDS) of SREBP and FOXO were cloned into the pAc5.1 vector and co‐transfected into *Drosophila* Schneider 2 (S2) cells. We supplemented the S2 cell culture medium with 10% PBS to simulate starvation, while the control group received 10% Fetal Bovine Serum (FBS). At the same time, cells under normal culture conditions were treated with 20‐hydroxyecdysone (20E) to mimic the late‐pupal stage, with ethanol added as the control. In the pAc5.1‐FOXO transfected group, S2 cells showed a significant increase in luciferase activity in response to PBS and 20E treatment compared to FBS and ethanol controls. In contrast, the pAc5.1‐SREBP group showed no significant changes in luciferase activity (Figure 5f,g). These results indicate that FOXO significantly enhances the regulation of miR‐375‐3p under 20E stimulation and starvation conditions.

To investigate FOXO's in vivo transcriptional regulation of miR‐375‐3p during starvation and pupal metamorphosis, we designed two ChIP‐qPCR primers targeting predicted FOXO binding sites from the Jaspar database (Figure S8e). Experimental groups included starvation vs. feeding conditions and early vs. late pupal stages. Results showed no significant changes in FOXO binding at the first binding site across all conditions. However, FOXO binding to the second site was significantly enhanced under starvation and in the late pupal stage (Figure 5h,i). EMSA confirmed that the second binding site interacted with purified FOXO recombinant protein (Figure 5j). Mutation of this site largely reduced luciferase activity linked to FOXO and the promoter region (Figure 5k). These findings suggest that FOXO binds to the sequence TAAAAACAC of miR‐375‐3p promoter, and its activity was enhanced under pupal metamorphosis and starvation conditions.

To test whether FOXO could regulate downstream immune responses and oxidative stress through miR‐375‐3p, we knocked down *FOXO* during the pupal stage and under starvation conditions, and performed a rescue experiment using miR‐375‐3p mimics. Under *E. coli* treatment, colony counts in both the pupal stage and starvation conditions decreased after injection of *dsFOXO*, subsequent addition of the miR‐375‐3p mimic partially restored those counts (Figure S9a). Under *S. aureus* treatment, colony counts in both the pupal stage and starvation conditions decreased after *dsFOXO* injection. However, only in the starvation condition did the subsequent addition of mimics‐375‐3p lead to a recovery, with no significant effect observed in the pupal stage (Figure S9b). This difference may be attributed to the distinct infection and defense mechanisms of Gram‐negative and Gram‐positive bacteria. Furthermore, enzyme activity assays of CAT and T‐AOC showed that *FOXO* knockdown during the pupal stage and under starvation conditions led to a significant decrease in antioxidant capacity. The addition of mimics‐375‐3p resulted in a partial recovery of this decline (Figure S9c,d).

### 2.8 FOXO Responds to Hormonal Signaling to Regulate miR‐375‐3p Expression

FOXO transcription factors are tightly regulated by signaling pathways, particularly the IIS and 20E pathways [40, 41]. We aimed to investigate whether FOXO responds to the induction of these two hormones during pupal stage and starvation conditions, and whether FOXO mediates the regulation of downstream miR‐375‐3p by these hormones. qPCR results showed that during the pupal stage, 20E signaling had a dominant effect over insulin, regulating miR‐375‐3p expression (Figure S10a,b). However, during feeding and starvation, both 20E and insulin regulated miR‐375‐3p expression via FOXO (Figure S10c–f).

Immunofluorescence analysis was performed to examine the nuclear translocation of FOXO. Consistent with the qPCR results, during the eclosion period, FOXO primarily responded to the activation of the 20E signaling pathway, enabling its nuclear entry to regulate miR‐375‐3p. Under starvation conditions, the inhibition of the insulin signaling pathway combined with the activation of the 20E signaling pathway similarly induced FOXO to translocate to the nucleus and regulate downstream genes (Figure S11). These findings underscore that 20E and insulin modulate FOXO activity during the pupal stage, feeding, and starvation through distinct modes, acting either independently or synergistically.

### 2.9 Energy Deficiency Induced miR‐375 High Expression is Conserved in Insects

To verify whether the high expression of miR‐375‐3p induced by energy deficiency is conserved, we selected two insect species for further investigation: the hemimetabolous insect, *Apolygus lucorum*, and the holometabolous insect, *Drosophila melanogaster*. The *A. lucorum* exhibited nearly 100% mortality after 24 h of starvation, and the *D. melanogaster* showed nearly 100% mortality after 2 days of starvation. Therefore, we measured miR‐375‐3p expression in *A. lucorum* at 0, 3, 6, 9, and 12 h, and in *D. melanogaster* at 0, 6, 12, and 24‐h post‐starvation, as well as during the pupal stage. Under starvation conditions, the expression level of miR‐375‐3p was significantly increased in both *A. lucorum* and *D. melanogaster* (Figure S12a,b). Moreover, during the pupal stage, the expression of miR‐375‐3p in *D. melanogaster* was also significantly elevated (Figure S12 c). These results provide preliminary evidence that miR‐375‐3p may function as a conserved regulatory axis under energy deficiency conditions.

## 3 Discussion

**FIGURE 6:**
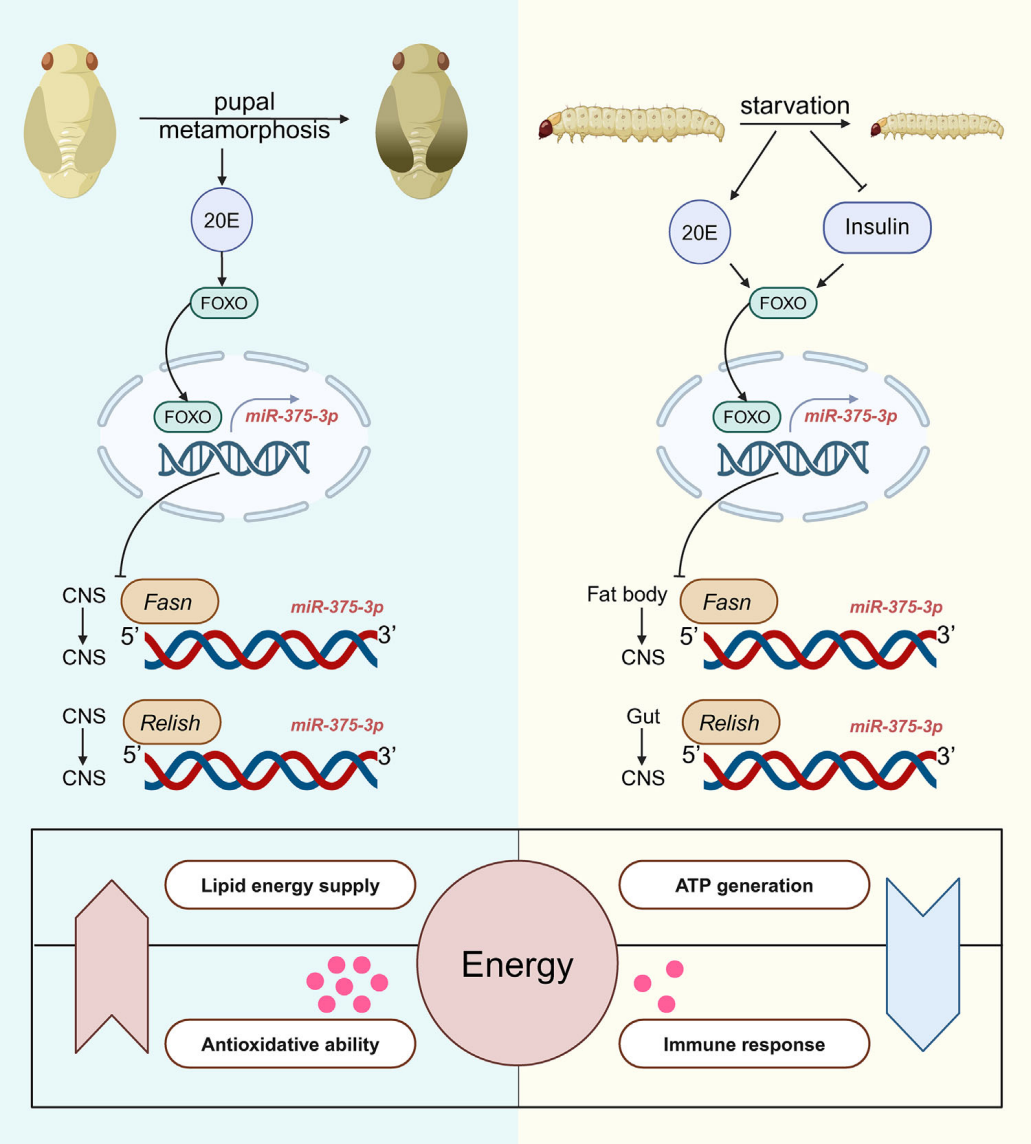
Summary model of the FOXO‐miR‐375 transcriptional axis regulates energy distribution to ensure survival under energy deficient conditions. During pupal metamorphosis, 20E is highly expressed, and during starvation, 20E remains elevated while insulin levels are suppressed. These hormonal changes trigger FOXO nuclear translocation, enabling it to bind to the promoter region of miR‐375‐3p and enhance its expression. Elevated miR‐375‐3p subsequently suppresses the expression of its downstream target genes, FASN and Relish. This upregulation of miR‐375‐3p promotes lipid droplet breakdown to supply energy, leading to reduced ATP availability. Additionally, energy is prioritized for antioxidant responses over immune reactions, ensuring the organism's survival under energy deficiency conditions.

The efficient supply and distribution of energy are fundamental for the survival and proper functioning of all living organisms [42, 43]. Throughout evolutionary history, energy deficiency has been a recurring challenge, driving the development of adaptive mechanisms to ensure survival under resource‐limited conditions [44, 45]. Insects, which constitute 75% of all animal species and are represent one of the most diverse and successful animal group  in the animal kingdom [46, 47], offer valuable models for understanding energy allocation strategies. In this study, using *T.castaneum* as a model organism, we reveal that miR‐375‐3p plays a pivotal role in regulating energy allocation under two energy deficient conditions, pupal metamorphosis and starvation. Pupal metamorphosis induces an upregulation of 20E, while starvation promotes 20E levels and suppresses insulin levels. The key transcription factor FOXO integrates the signaling cascades triggered by these hormonal changes, translocated to the nucleus, and upregulates the expression of miR‐375‐3p. Furthermore, miR‐375‐3p directly targets *FASN* and *Relish*, and indirectly affects *CAT* levels, thereby ensuring the balance between growth and maintenance under conditions of energy deficiency (see model in Figure 6).

In our previous study, we performed sequencing of miRNA expression at different stages of *T. castaneum* and validated the reliability of the sequencing results [32, 48]. Further analysis revealed that miR‐375‐3p exhibited the most significant expression changes between early and late pupal stages. Interestingly, although endogenous miR‐375‐3p is upregulated during specific pupal stages, injection of miR‐375‐3p mimics resulted in a subset of pupae failing to complete metamorphosis. Previous studies indicate that miRNAs exhibit threshold like regulation of their target mRNAs, showing strong repression below and hypersensitive responses near the threshold [49]. Consequently, both insufficient and excessive miRNA expression can cause abnormal phenotypes. Overaccumulation of small RNAs may also saturate components of the RNAi machinery, such as Exportin‐5, leading to non‐physiological toxicity [50, 51].In insects, there are also intuitive examples. 20E is an essential hormone for larvae to pupate. Early injection of 20E can induce larvae to pupate prematurely, but excessive injection of 20E can lead to larval death [52]. These findings may also help explain why overexpression of miR‐375 led to incomplete eclosion in some beetles observed in our study. The function of miR‐375 was initially discovered in diabetes, where it serves as a key regulator of insulin secretion and glucose homeostasis [53, 54]. Previous studies have also shown that weight loss surgery and short‐term very low‐calorie diets lead to elevated levels of miR‐375‐3p [55, 56]. Therefore, we hypothesize that the fluctuation of miR‐375 during pupation is caused by energy deficiency, resulting from the high energy consumption associated with organ remodeling during pupation and the lack of energy intake. Subsequent starvation treatment confirmed that miR‐375‐3p is highly expressed under energy deficiency conditions, and its knockdown leads to *T. castaneum* mortality. It is noteworthy that miR‐375‐3p undergoes an early transient elevation, followed by stage‐dependent or feedback‐mediated reduction. This pattern may reflect fine‐tuned temporal regulation during development, in which miR‐375‐3p initially reaches a level sufficient to suppress its targets during the early pupal phase but subsequently returns to a physiological baseline to prevent excessive repression. Similar temporal decay phenomena have been widely observed in vertebrate and invertebrate developmental contexts, where miRNAs exhibit carefully orchestrated, stage‐specific expression patterns driven by both intrinsic decay programs and feedback regulation [57, 58].

To explore whether this regulatory mechanism is evolutionarily conserved, we examined miR‐375‐3p expression in two additional insect species: the hemimetabolous insect *A. lucorum* and the holometabolous insect *D. melanogaster*. In both species, starvation significantly increased miR‐375‐3p expression, and in *D. melanogaster*, a marked elevation of miR‐375‐3p was also observed during the pupal stage. These results provide preliminary evidence supporting a conserved role of miR‐375 in mediating energy deficiency responses across different insect taxa. Notably, miR‐375 is also deeply implicated in energy metabolism in mammals. In human and mouse studies, miR‐375 has been identified as a key biomarker for both type 1 and type 2 diabetes and is known to regulate pancreatic β‐cell function and glucose homeostasis [59, 60]. Moreover, chronic energy restriction is a major contributing factor to type 2 diabetes, and recent findings show significant alterations in liver and β‐cell metabolic indicators under carbohydrate and energy restricted conditions, with miR‐375 serving as a relevant molecular indicator [61]. These cross‐species observations further support the idea that miR‐375 participates in a fundamentally conserved mechanism governing physiological adaptation to energy shortage. Taken together, our results suggest that miR‐375‐3p is closely associated with energy metabolism in both insects and mammals and exhibits a consistent regulatory pattern under energy‐deficient conditions. We propose that changes in miR‐375‐3p expression may represent an evolutionarily conserved adaptive mechanism that enables organisms to cope with metabolic stress during periods of limited energy availability.

Using dual luciferase assay, immunoprecipitation, fluorescence in situ hybridization RNA‐seq and bioinformatics analysis, our study demonstrated that miR‐375‐3p could act on the *FASN* and *Relish* to inhibit their transcription, respectively. FASN, an essential cytosolic enzyme in De novo lipogenesis (DNL) pathway, catalyzes the biosynthesis of saturated fatty acids from acetyl‐CoA (AcCoA) and malonyl coenzyme A (malonyl‐CoA) [62]. During the development of *Drosophila* embryos and larvae, the mRNA expression of several lipogenic enzymes, including *FASN*, is correlated with TAG levels [63, 64]. Moreover, *Drosophila* with mutations in *FASN1* and *FASN2* store less TAG during both the larval and adult stages, suggesting that FASN is a key enzyme in TAG storage [65]. TAG levels are tightly regulated during *Drosophila* development, with a significant accumulation of TAG being a characteristic of larval growth [63, 64]^)^. In addition, under conditions like excess nutrition, growth factor stimulation, obesity, diabetes, fatty liver diseases, or cancer, DNL is significantly elevated, and the mRNA expression of *FASN* positively correlates with elevated lipogenesis [66, 67, 68, 69]. Therefore, FASN regulates energy allocation by influencing fatty acid synthesis in response to energy deficiency. Currently, NF‐κB factors/Relish are predominantly recognized for their role in transcriptionally activating antimicrobial peptides (AMPs) in insects and regulating downstream cytokines in humans through their transactivation domain (TAD) [70]. However, AMP production, while crucial for immune defense, is metabolically costly. It is energy‐intensive, and overactivation can lead to host damage, including membrane disruption, tissue inflammation, and immune imbalance [71, 72]. Furthermore, Relish regulates lipid metabolism in *Drosophila* by downregulating *bmm* controlling fasting‐induced lipolysis [35]. It can be inferred that under conditions of energy deficiency, the body promotes fatty acid breakdown and suppresses immune responses through the low expression of *Relish*. In addition to immune responses, oxidative stress also plays a crucial role in the balance of energy allocation [6]. Our RNA‐seq and experimental data both indicate that miR‐375‐3p promotes the expression of CAT, an important antioxidant enzyme [73], thereby enhancing antioxidant capacity. Under energy deficiency, miR‐375‐3p upregulated, inhibiting *FASN* and *Relish*, promoting *CAT*, suppresses lipogenesis, and enhances lipolysis while reducing immune responses and boosting antioxidant capacity. Interestingly, this mechanism resembles a form of simple economic behavior, where energy functions like currency: in the absence of income, organisms reduce expenditures on fatty acid synthesis, are forced to withdraw reserves through fatty acid breakdown, and prioritize spending on essential biological functions like antioxidant defense, while cutting back on “luxuries” such as immune responses, which require high metabolic energy.

It is well known that, in addition to the trade‐offs between overall functions, there are also varying priorities in energy allocation across different organs [74]. For example, during starvation, polyploid tissues such as the salivary glands and fat body cease growth [75], while glycogen derived from fat body helps maintain trehalose levels in the hemolymph [76]. As a result, nutrient reserves are primarily directed toward the central nervous system (CNS) to support its ongoing function and growth. This conclusion is further supported by our study on the expression patterns of FASN and Relish under different physiological conditions. During feeding, *FASN* is predominantly expressed in the fat body, while *Relish* shows high expression in the gut. However, during starvation and pupal metamorphosis, both genes are significantly upregulated in the central nervous system (CNS). Interestingly, not only lipid metabolism, knockdown of miR‐375‐3p leads to significant alterations in ATP levels. Under feeding conditions, miR‐375‐3p knockdown results in increased ATP content across the whole organism, including the gut, CNS, and fat body, suggesting enhanced energy metabolism. In contrast, during starvation and pupal metamorphosis, miR‐375‐3p knockdown causes a significant rise in ATP levels in the CNS, while ATP levels decrease in the gut and fat body. This suggests that miR‐375‐3p plays a pivotal role in regulating energy distribution, aiding in the coordination of lipid metabolism and ATP production across various tissues.

Notably, our study further demonstrates a negative correlation between cellular energy status and miR‐375‐3p expression. Numerous reports have shown that miR‐375 is frequently downregulated in tumors such as hepatocellular carcinoma [77], gastric cancer [78], breast cancer [79] and esophageal cancer [80]. In malignancies, lipid metabolism is commonly reprogrammed to meet the high energy demands and rapid proliferation of cancer cells: de novo lipogenesis is upregulated, and FASN is often overexpressed to ensure an abundant supply of fatty acids for membrane biogenesis, ATP production, and signaling lipid synthesis [81]. Although our experiments were conducted under energy deprived conditions, rather than the energy rich, lipogenesis driven environment typical of tumors, we likewise observed a marked upregulation of miR‐375‐3p. This suggests that shifts in cellular energy status trigger miR‐375‐3p via a negative feedback mechanism. Moreover, it allows us to hypothesize that miR‐375‐3p's role in regulating lipid metabolism may constitute a broadly conserved mechanism across species.

In addition to miR‐375‐3p's role in energy allocation, we explored potential upstream transcriptional regulators. We identified two potential factors, with FOXO showing binding activity to the miR‐375‐3p promoter, unlike SREBP. SREBP binds to the FASN promoter only during feeding, with binding disappearing after fasting [82, 83, 84]. This suggests FOXO mediates miR‐375‐3p transcription under energy‐scarce conditions, while SREBP primarily regulates lipid metabolism during feeding. Previous studies have shown that FOXO cooperates with the insulin‐like peptide target CREB during fasting in mammals and is directly or indirectly inhibited by insulin through protein kinase B [85, 86, 87]. Additionally, 20E upregulated FOXO during non‐feeding stages to reduce TAG levels in fat bodies [33].

In summary, our study identifies a hormone‐responsive FOXO–miR‐375‐3p transcription axis as a central regulator of systemic energy allocation under energy deficient conditions. This axis links FOXO‐mediated hormonal inputs to miR‐375‐3p's post‐transcriptional control of key metabolic and stress‐response genes, redirecting resources toward developmental and survival pathways under energy deficient conditions. This mechanism could also be extended to different species and had the potential to become a target for the prevention and treatment of metabolic diseases and tumors.

## 4 Materials and Methods

### 4.1 Insect and Bacteria

The Georgia‐1 strain of *T. castaneum* was reared on wheat flour containing 5% brewer's yeasts under constant conditions at a temperature of 30°C and relative humidity of 40% with a photoperiod of 14:10 h (light: dark) in a climatic chamber [88]. Flies were obtained from the Bloomington *Drosophila* Stock Center, all flies were raised on maize malt molasses food in a light‐dark (12‐hr cycle) incubator at 25°C and 60% humidity. *A. lucorum* were collected from experimental fields at the Hebei Langfang Station of the Chinese Academy of Agricultural Sciences. Insect rearing was carried out in environmental chambers set at a constant temperature of 28 ± 1 °C, with a relative humidity of 70 ± 5%, and a photoperiod of 14:10 (light: dark). We used transparent plastic containers for rearing, each of which could accommodate approximately 200 nymphs or 150 adults. Two types of microorganisms used here included a Gram‐positive bacterium (G+) (*S. aureus*) and a Gram‐negative bacterium (G‐) (*E. coli*). All bacteria were cultured in LB medium at 37°C.

### 4.2 Bioinformatic Analysis of miR‐375‐3p

16 sequences of miR‐375‐3p from different species were obtained from miRbase [89], and these sequences were aligned with ClustalW in MEGA v7 [90]. Multiple sequence was visualized by ESPript 3.0 (http://espript.ibcp.fr/ESPript/cgi‐bin/ESPript.cgi)

### 4.3 Small RNA Sequencing

The purified small RNAs (15–30 bases, bp) were ligated to a pair of adaptors on their 5′ and 3′ ends (Illumina, San Diego, CA, USA). The small RNAs were amplified using the adapter primers for 17 cycles, and 70–90 bp fragments (small RNA plus adaptors) were excised from gels. Then, the purified DNA fragments were used for cluster generation on Illumina Cluster Station and sequenced on Illumina GAIIx following the vendor's instructions. Raw sequencing reads were obtained using Illumina Sequencing Control Studio software version 2.8 (SCS v2.8) following real‐time sequencing image analysis and base‐calling by Illumina Real‐Time Analysis version 1.8.70 (RTA v1.8.70) [91].

### 4.4 Double‐Stranded RNAs Synthesis

RNAiwas performed as previously described [92]. Briefly, double‐stranded RNAs (dsRNAs) specific for target gene were synthesized with the TranscriptAid T7 High Yield Transcription Kit (Fermentas, Vilnius, Lithuania) according to the instruction.

### 4.5 Quantitative PCR Assays of Genes and miRNAs

qRT‐PCR was performed on an ABI7900 system (Applied Biosystems; Life Technologies). The *T. castaneum* ribosomal protein S3 (rps3, GenBank accession numbers CB335975) gene served as an internal control [88] The relative expression levels of miRNAs were calculated according to the formula 2^−ΔΔCt^ [93], and U6 snRNA was used as an internal reference for miRNA detection. Three biological replicates were performed. Data were compared using one‐way ANOVA and Duncan's multiple range test; *p* < 0.05, *n* = 3.

### 4.6 RNA‐seq

The gut of late pupa injected with antagomir‐NC and antagomir‐375‐3P were collected, and each sample contained 80 guts. Three independent replicates were performed for each group. Total RNA was isolated, and RNA quality was confirmed by agarose gel electrophoresis. cDNA libraries were prepared in accordance with Illumina protocols (Yingzi Gene Technology, Wuhan, China). Index codes were added to attribute sequences to each sample. Clean reads were obtained by filtering the low‐quality sequences. These reads were mapped to the reference genome of *T. castaneum* (ftp://ftp.ncbi.nlm.nih.gov/genomes/all/GCF/000/002/335/GCF_000002335.3_Tcas5.2/). StringTie software (v2.2.1) was used to calculate the fragments per kilobase million values of genes. DEGs were analyzed using EdgeR software. The DEGs with a fold change of p < 0.01 were selected. Gene enrichment analysis was performed by KEGG signaling pathway analysis. Additionally, RNA‐seq data were deposited in the NCBI Sequence Read Archive Database (accession number: PRJNA1182992)

### 4.7 miRNA Mimics and Antagomir Treatment in Vivo

miRNA mimics and antagomir were obtained from GenePharma (Shanghai, China). Mimics and antagomir of miR‐375‐3p were non‐natural double‐stranded RNA fragments mimicking. It was synthesized according to its mature sequence5’ UUUGUUCGUGUGGCUCGAGUUA3’. The sequence of a Caenorhabditis elegans (mimics‐NC sense:UUCUCCGAACGUGUCACGUTT antasense: ACGUGACACGUUCGGAGAATT, antagomir‐NC sense:CAGUACUUUUGUGUAGUACAA)was used as a negative control. The mimics and antagomir were injected into *T. castaneum* body cavities at a dose of 400 µm in a volume of 200 nL.

### 4.8 RNA Interference in Vivo

To knock down related genes, *T. castaneum* were each injected with a dose of 200 ng in a volume of 200 nL (World Precision Instruments, USA). Control beetles were injected with equal amounts of *dsEGFP*. The effect of RNAi on the relative mRNA expression levels was investigated by qPCR after injection. The primers employed for RNAi are provided in Dataset S6.

### 4.9 Localization of miRNA by FISH

The whole bodies of *T. castaneum* were fixed in 4% paraformaldehyde overnight and embedded in paraffin. Histological sections were prepared and incubated with probes using a fluorescence in situ hybridization kit (GefanBio, Shanghai, China). The Cy3‐labeled miR‐375‐3p probe was designed and synthesized by GefanBio (Dataset S6).

### 4.10 Immunofluorescence

Frozen sections or tissues were fixed in 4% (vol/vol) paraformaldehyde, rinsed with PBS‐T [0.3% (vol/vol) Triton X‐100 in PBS], kept in 3% (wt/vol) BSA‐PBS‐T for 1 h, incubated overnight with primary antibody rabbit Anti‐Relish Antibody (1:100; RayBiotech), rabbit Anti‐FASN (1:100; Bioworlde) and rabbit Anti‐FOXO Antibody (1:500; GenScript). Antibody incubated 2 h with FITC labeled secondary antibody and Cy3 labeled secondary antibody (goat anti‐rabbit; Servicebio), stained with DAPI (Servicebio), then imaged under a fluorescence microscope (Olympus BX43) and a confocal laser scanning microscope (Nikon Ti). The mean fluorescence intensity was analyzed using ImageJ software (https://imagej.nih.gov/ij/docs/index.html).

### 4.11 In Vitro Luciferase Reporter Gene Assays

The pAc5.1/V5‐HisA expression vector (Thermo) was used for constructing the recombinant plasmids. And the luciferase coding sequence was subcloned into pAc5.1/V5‐HisA to generate pAc‐luc as previous [94]. To produce exogenous FOXO and Srebp in *Drosophila* S2 cells, the CDS sequences of Foxo and Srebp were amplified and cloned into pAc5.1/V5‐HisA vector to generate pAc‐FOXO and pAc‐SREBP plasmids using ClonExpress II One Step Cloning Kit (Vazyme, China). The sequences of FASN and Relish with or without miR‐375‐3p binding sites were amplified by PCR and cloned into pAc‐luc to generate pAc‐luc‐FASN, pAc‐luc‐FASN‐mut, pAc‐luc‐Relish and pAc‐luc‐Relish‐mut. We constructed the promoter region, containing about 1.5 kb upstream from miR‐375‐3p TSS site, into the pGL3‐Basic plasmid (Promega, USA). All construction ones were verified by Sanger sequencing. All primers used in this analysis were listed in Dataset S6. For miRNA target gene experiments, S2 cells were transfected with the mixed transfection complex contained either mimics‐miR‐375‐3p or mimics‐NC, either pAc‐Luc‐FASN or pAc‐luc‐FASN‐mut, either pAc‐luc‐Relish or pAc‐luc‐Relish‐mut and Renilla luciferase plasmid (pRL) for a total volume of 50 µL. Promega pRL was used to normalize the transfection efficiency. To explore whether FOXO and Srebp could regulate the promoter activity of miR‐375, we transfected pGL3‐miR‐375‐3p‐promoter, pAc‐FOXO and pAc‐SREBP into S2 cells. After transfection, cells were exposed to 20E (10^−6^ m) dissolved in ethanol, ethanol alone, as a simulation of pupal metamorphosis. Another group of cells was added with 10% FBS or PBS as a simulation of starvation. The luciferase activity was measured using the Dual‐Luciferase Reporter Assay System (Promega, USA) according to manufacturer's instructions and the Renilla luciferase activity was used as a control for normalization.

### 4.12 RIP Assays in Vivo

The RIP assays were performed using a RNA Binding Protein Immunoprecipitation Kit (Gene Create). The monoclonal antibody against Ago‐1 protein was developed in mice (Proteintech). Beetles were collected and homogenized in ice‐cold RIP lysis buffer, then stored at −80 °C overnight. Magnetic beads were pre‐incubated with 4 µg of Ago‐1 antibody (Proteintech) or with 5 µg of normal mouse IgG (Gene Create)), which was used as a negative control. Next, the frozen homogenates were thawed and centrifuged, and the supernatant was incubated overnight with the magnetic bead–antibody complex at 4 °C. Meanwhile,1/10 of the lysate was stored as “input” sample. The RNA in the immunoprecipitates and inputs d was purified and reverse‐transcribed into cDNA using the high‐capacity RNA‐to‐cDNA Kit (CWBIO). qPCR were performed using these cDNAs as templates to quantify the mRNA of predited gene. To normalize the relative expression levels, the supernatants of the RIP lysate (input) and the IgG controls were assayed for specificity of RNA–protein interactions. Approximately 40 beetles were microinjected with mimics‐375‐3p. A scrambled miRNA agomir was used as a negative control.

### 4.13 ChIP Assays in Vivo

A Pierce Magnetic ChIP Kit (Thermo) was used to obtain genomic interactions of transcription factors with chromosomal DNA. Briefly, tissues were dissected and sheared in Tissue Stabilizing Solution with Protease Inhibitor, fixed using 1% formaldehyde, treated with Tissue Lysis Buffer/ Protease Inhibitor and sonicated on wet ice. The targeted chromatin was pulled down by G bead‐antibody of interest. The protein‐DNA crosslinks were reverse transcribed, and the DNA was purified for downstream applications(qPCR). IgG of the same species as the antibody of interest was used as a negative control. RNAi‐mediated disruption of the protein of interest was used as a control. The fold enrichment represents three biological replicates. The first group compared 40 insects treated with starvation for 48 h and fed for 48 h, while the second group compared 40 early pupae and 40 late pupae.

### 4.14 Metabolic Assays in Vivo

Metabolic assays were performed as previously described [95]. Briefly, 50 beetles' intestines, 80 beetles' CNS and 30 beetles' fat bodies were homogenized in 1 mL PBST buffer (0.05% Tween 20) and immediately incubated at 70°C for 5 min to inactivate endogenous enzymes. The homogenate was centrifuged for 5 min at 13 000 g to remove fly debris, and the supernatant was used for the subsequent metabolic determination. TAG was quantified using a triglyceride determination kit (Jian Cheng) according to the manufacturer's protocol. ATP assay was quantified using a ATP Assay Kit (Beyotime). Metabolite measurement data are normalized to the total protein concentrations obtained by the BCA assay (Jian Cheng). The experiments were conducted with three independent biological replicates.

### 4.15 Enzyme Activity Assay in Vivo

Total antioxidant capacity(T‐AOC) and Catalase (CAT) assay were quantified using a Total antioxidant capacity assay kit (Jian Cheng) and a Catalase (CAT) assay kit (Jian Cheng) according to the manufacturer's protocol.

### 4.16 ROS Detection in Vivo

H2DCFH‐DA was diluted into PBS at a ratio of 1:1000 and added to frozen sections. Frozen sections were incubated for 30 min at 37 °C, and then washed 3x with PBS. Frozen sections were imaged using 488 nm excitation and 525 nm emission.

### 4.17 Rapid Amplification of cDNA Ends (RACE)

Total RNA of Drosha‐knocked‐down beetles was used to generate cDNA for 5’ RACE using the GeneRacer kit (Invitrogen) according to the manufacturer's instructions. The primers used for cDNA generation and PCR amplification are listed in Dataset S6. After cloning the amplified fragments, we analyzed their sequences by means of Sanger sequencing. For each miRNA, 12 clones were sequenced.

### 4.18 Expression and Purification of Recombinant Protein in Vitro

A DNA fragment encoding the CDS of FOXO was inserted into the pET‐28a (+) expression vector (Novagen, Darmstadt, Germany), respectively. The recombinant construct pET‐28a‐ FOXO was transformed into competent cells of Escherichia coli BL21 (DE3) (TransGene, Beijing, China) for protein expression. The pET‐28a (+) vector without insert fragment was also transformed into competent *E. coli* BL21 (DE3) cells that served as a control. The recombinant FOXO (rFOXO) was purified by affinity chromatography using ProteinIso Ni‐NTA Resin (TransGen Biotech, Beijing, China) under denatured conditions. The purified rFOXO was dialyzed in gradient urea‐Tris‐buffered saline (TBS) glycerol buffer. The rFOXO was detected by 12.5% SDS‐polyacrylamide gel electrophoresis (SDS‐PAGE) and visualized with Coomassie brilliant blue R‐250. The concentration of rTcFOXO was quantified with the total protein quantitative assay kit (Nanjing Jiancheng Bioengineering Institute, Nanjing, China).

### 4.19 EMSA Assays in Vitro

A 6% TBE gel (Solarbio) was prepared according to the kit instructions, and the EMSA experiment was conducted following the provided protocol (Thermo). Briefly, the DNA probe and purified protein were incubated at room temperature for 20 min in the presence of a binding buffer solution. After incubation, a non‐denaturing and non‐reducing protein loading buffer was added for sample preparation and electrophoresis. The gel was run at a constant voltage of 100 V for 60 min at 4°C. Following electrophoresis, the gel was placed in a clean container and stained with 1 × SYBR Green EMSA Nucleic Acid Dye (prepared by diluting the dye 1:20 000 in 0.5 × TBE buffer). The gel was covered with the dye solution and incubated in the dark at room temperature for 20 min. After staining, the dye solution was discarded, and the gel was washed three times with deionized water in the dark. Finally, the gel was imaged and the results were stored using a gel imaging system.

### 4.20 Statistical Analysis

Data were presented as mean ± SEM of three independent biological replicates, unless otherwise noted. Survival curves were generated and analyzed by Log‐rank (Mantel‐Cox) test using Prism 9. Significant differences between treatments/groups were analyzed using a student's t‐test, one‐way ANOVA, or two‐way ANOVA: ^*^ *p* < 0.05; ^**^ *p* < 0.01; ^***^ *p* < 0.001; ^****^ *p* < 0.0001. Different letters represent different levels of significance, while ns represents no significance. The experiments were randomized. The experiment was conducted using a single blind method.

## Conflicts of Interest

The authors declare no conflicts of interest.

## Supporting information




**Supporting File 1**: advs74132‐sup‐0001‐SuppMat.docx.

**Supporting File 2**: advs74132‐sup‐0002‐Data.zip.

## Data Availability

The data that support the findings of this study are available from the corresponding author upon reasonable request.
