# Supplementary material for: MicroRNA‐375‐3p Targets Fatty Acid Synthase and Relish to Regulate Energy Allocation During Pupal Metamorphosis and Starvation
Source: Adv Sci (Weinh). 2026 Feb 12;13(20):e13486. doi: 10.1002/advs.202513486 (PMC13067826; doi:10.1002/advs.202513486)
Supplement: Supplementary file 1 — Supporting File 1: advs74132‐sup‐0001‐SuppMat.docx. [file ADVS-13-e13486-s002.docx]

**Supporting Information for**

MicroRNA-375-3p targets Fatty acid synthase and Relish to regulate energy allocation during pupal metamorphosis and starvation

Peng Chen^a^, Meiqi Cheng^a^, Jianhui Wang^a^, Jing Tang^a,b^, Xiaoqiao Huang^a^, Yusi Li^a^, Huiling Zhou^a^, Ling Zhang^a^, Yi Dong^a^, Chengjun Li^a, *^ and Bin Li^a,*^

^a^College of Life Sciences, Nanjing Normal University, Nanjing 210023, China

^b^Anhui Finance & Trade Vocational College, Anhui 230031, China

* Chengjun Li and Bin Li

Email: [lcj3314@163.com](mailto:lcj3314@163.com), or libin@njnu.edu.cn

Supporting Information Text

Fig. S1.


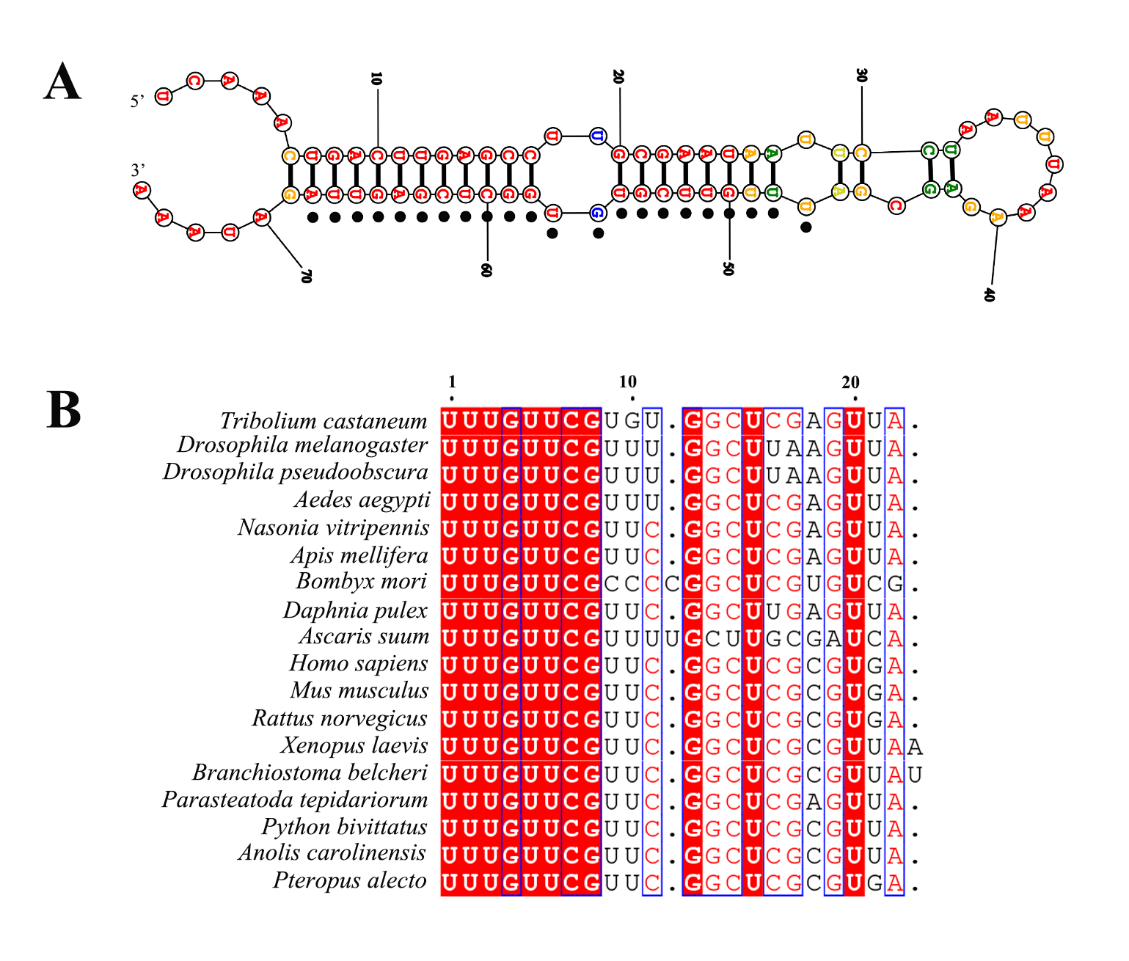


Fig. S1 Sequence feature of tca-miR-375-3p. (a) The predicted hairpin structure of tca-miR-375-3p, black dots indicated mature tca-miR-375-3p. (b) Multiple alignment of miR-375-3p in different species.

Fig. S2.


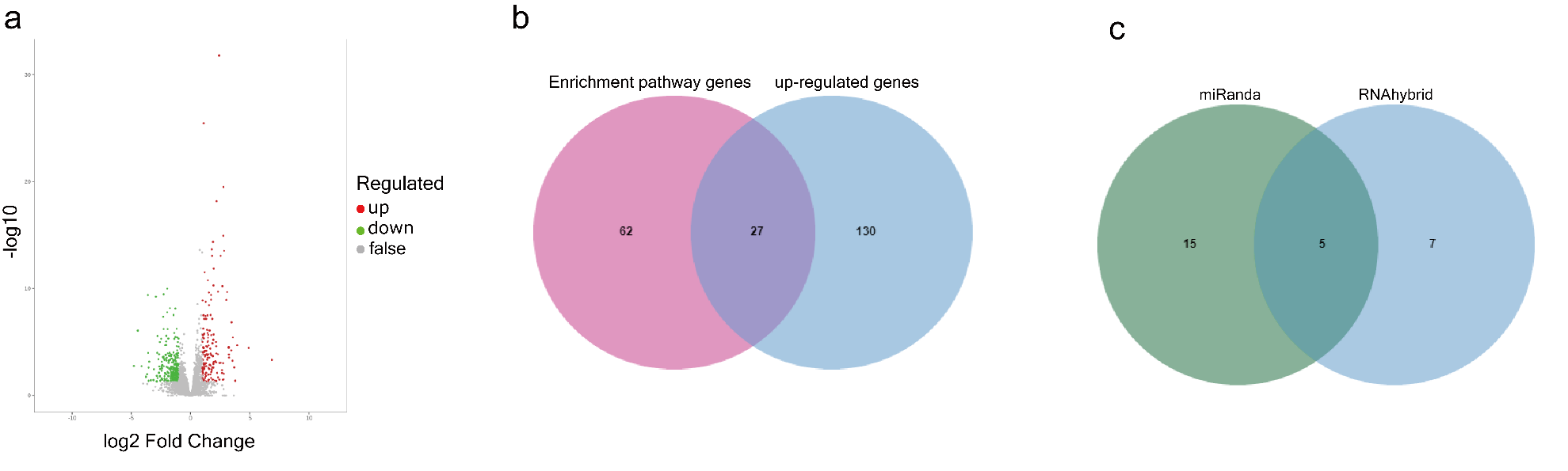


Fig. S2 a A volcano plot of the transcriptome shows significantly altered genes, with red indicating upregulated genes and green indicating downregulated ones. b Venn diagram of enriched pathways and upregulated genes. c Venn diagram of genes predicted by miRanda and RNAhybrid.

Fig. S3.


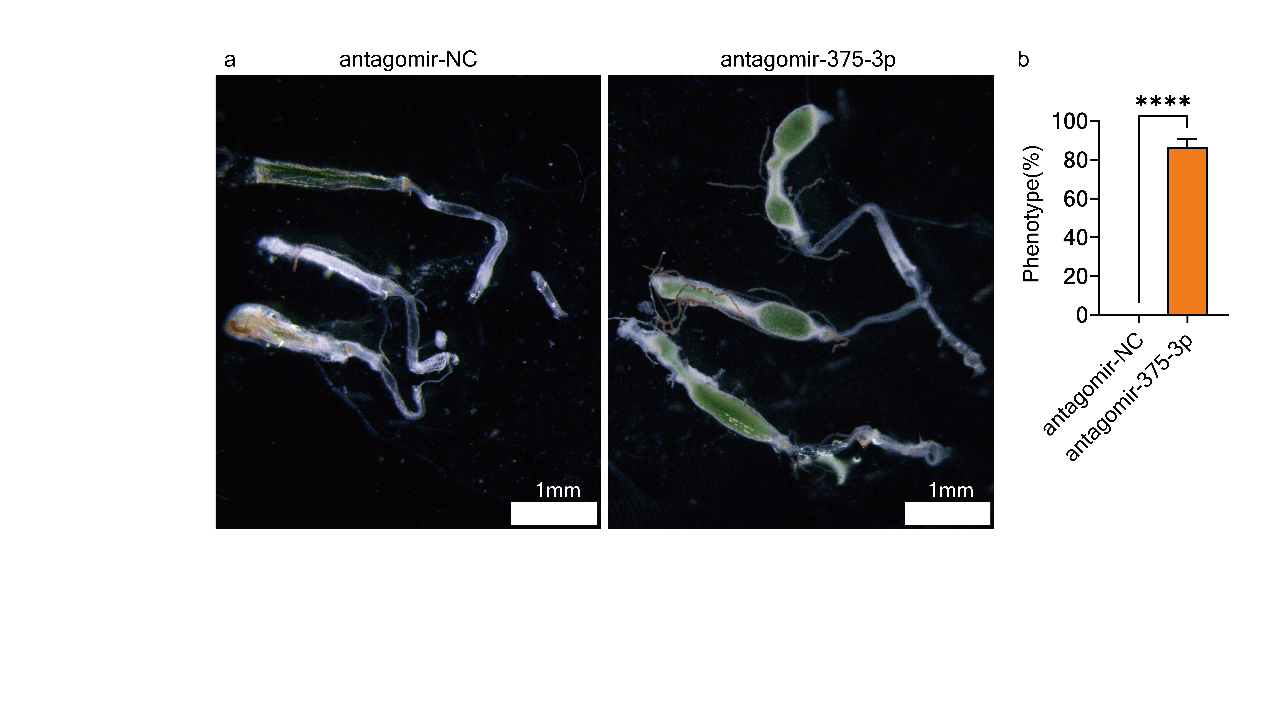


Fig. S3 Phenotype of gut after knocking down miRNA

a After the miRNA knockdown, the intestinal tract was filled with content. b Among three experimental groups, each consisting of 15 individuals, the intestinal phenotype rate of *T.castaneum* was observed and recorded. (Error bars represent SEM. ****p < 0.0001)

Fig. S4

Fig. S4 *FASN* and *Relish* regulates the growth and development of *T.castaneum* by affecting lipid metabolism

a Expression levels of *FASN* and *Relish* in the beetles based on qPCR. b The beetles were treated feed or starved for 48 hours. Whole bodies of beetles were homogenized in PBST solution, and then the contents of TAG. c Weigh gain rate of beetles treated with feed or starved for 48 hours. d Length gain rate of beetles treated with feed or starved for 48 hours. e Body weight of different treatment groups (1 point for every 5 insects) f Body length of different treatment groups. g Lipid droplet staining of beetles treated with feed or starved for 48 hours. h-k Expression of lipid metabolism related genes after knockdown or overexpression of mir-375-3p. (Error bars represent SEM. ****p < 0.0001, **p < 0.01, *p < 0.05 and ns means no significance, Student's t-test).

Fig. S5


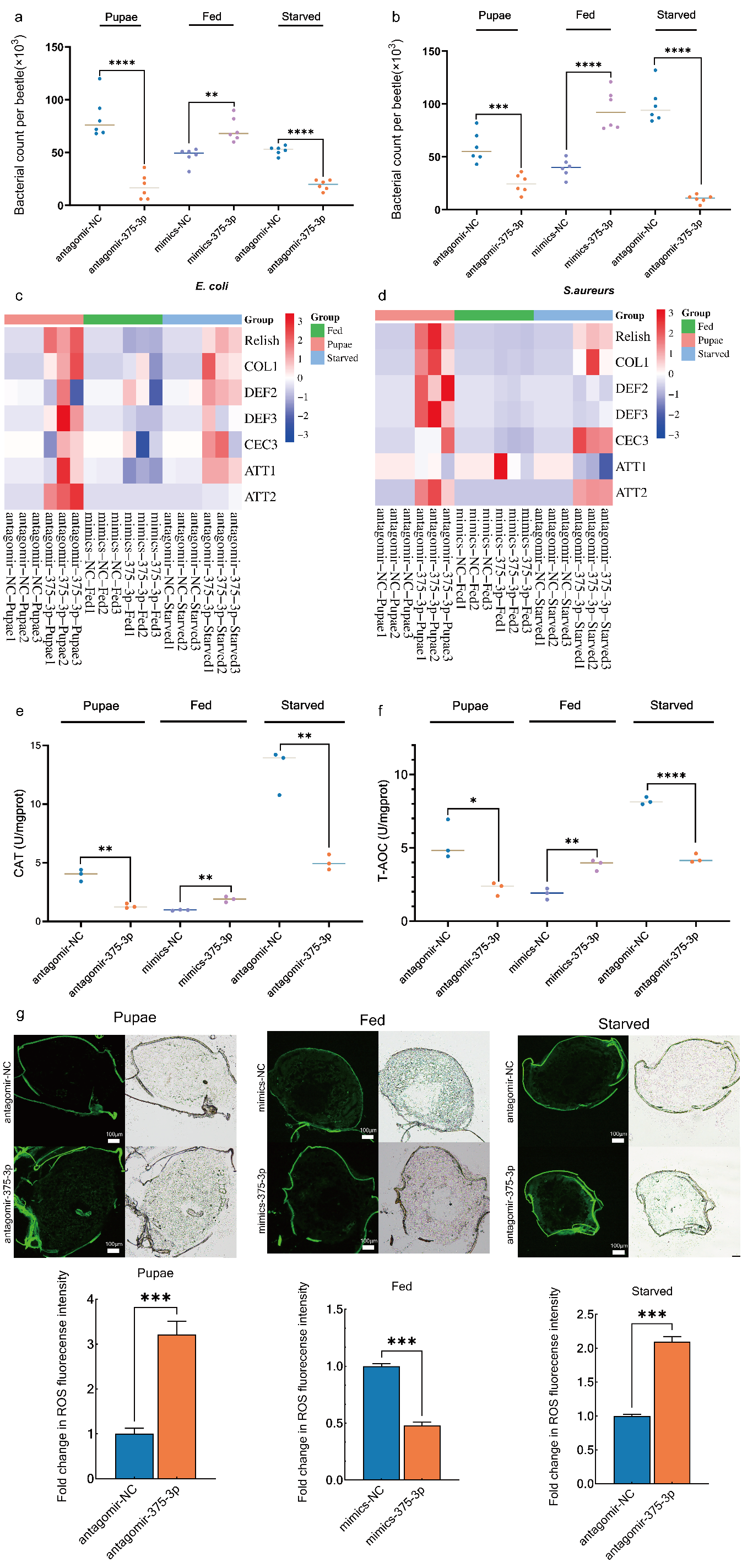


Fig. S5. mir-375-3p inhibits immune response and oxidative stress during pupal metamorphosis and starvation

a-b The bacterial count of the beetles infected with *E. coli* and *S. aureurs* after injected with antagomir-NC and antagomir-375-3p during pupal and starvation stage while injected with mimics-NC and mimics-375-3p during feeding stage. c-d AMP gene expression upon challenge with *E. coli* and *S. aureurs* after knockdown or overexpression of mir-375-3p. e-f Activities of CAT and T-AOC after injected with antagomir-NC and antagomir-375-3p during pupal and starvation stage while injected with mimics-NC and mimics-375-3p during feeding stage. g ROS levels displayed by H2DCFH staining after injected with antagomir-NC and antagomir-375-3p during pupal and starvation stage while injected with mimics-NC and mimics-375-3p during feeding stage. The bar graph represents the quantification of ROS levels. (Error bars represent SEM. ***p < 0.001, **p < 0.01 *p < 0.05 and ns means no significance, Student's t-test).

Fig. S6


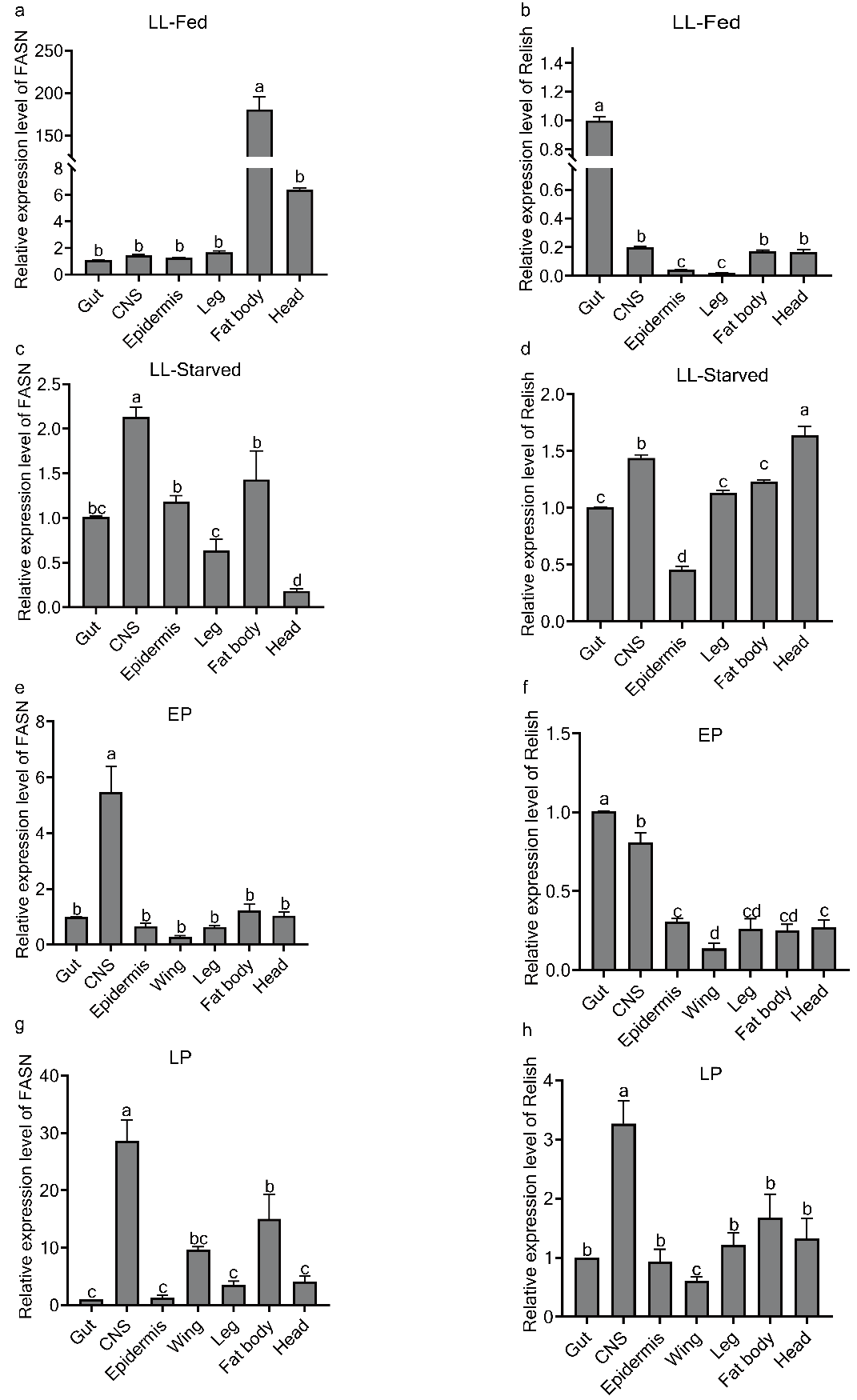


Fig S6 Organizational expression profiles of *FASN* and *Relish*

The expression pattern of *FASN* and *Relish* in different tissues. Tissues include: gut, CNS, epidermis, wing, leg, fat body and head. The significant differences among different treatments are indicated by lowercase letters above each bar (one-way ANOVA followed with Tukey’s test, different letters indicate significant differences within treatments).

Fig. S7


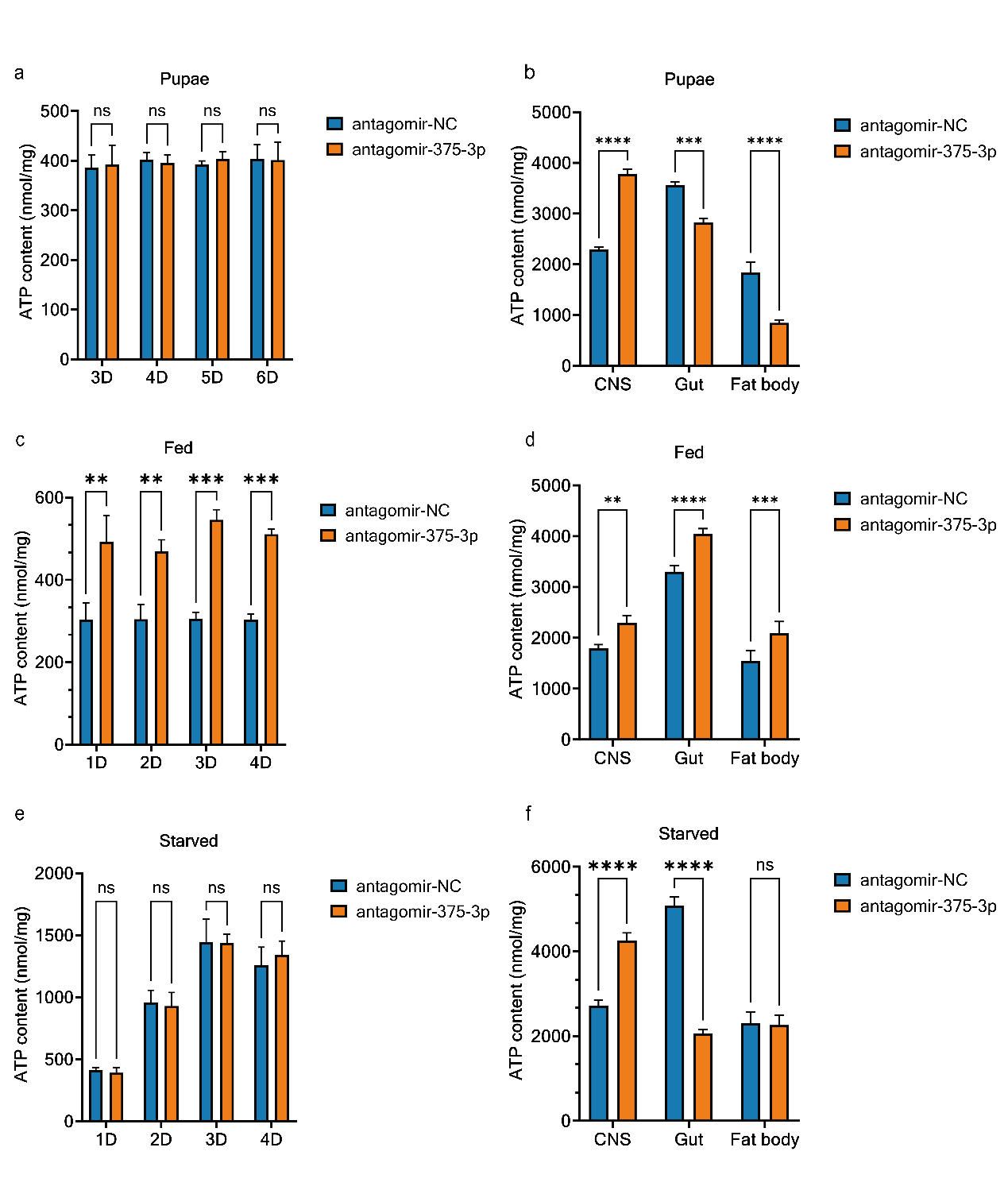


Fig. S7. miR-375-3p maintains ATP homeostasis in a tissue-specific manner during starvation and pupal metamorphosis.

a Knockout of miR-375-3p increases overall ATP content during the feeding period. b Knockout of miR-375-3p increases ATP content in the CNS, gut, and fat body during the feeding period. c Knockout of miR-375-3p did not affect the basal ATP content in beetles during the starvation period. d Knockout of miR-375-3p increases ATP content in the CNS while decreases ATP content in the gut during the starvation period. e Knockout of miR-375-3p did not affect the basal ATP content in beetles during the pupal metamorphosis. F Knockout of miR-375-3p increases ATP content in the CNS while decreases ATP content in the gut and fat body during the pupal metamorphosis. (Error bars represent SEM. ****p < 0.0001, ***p < 0.001 **p < 0.01 and ns means no significance, Student's t-test).

Fig. S8


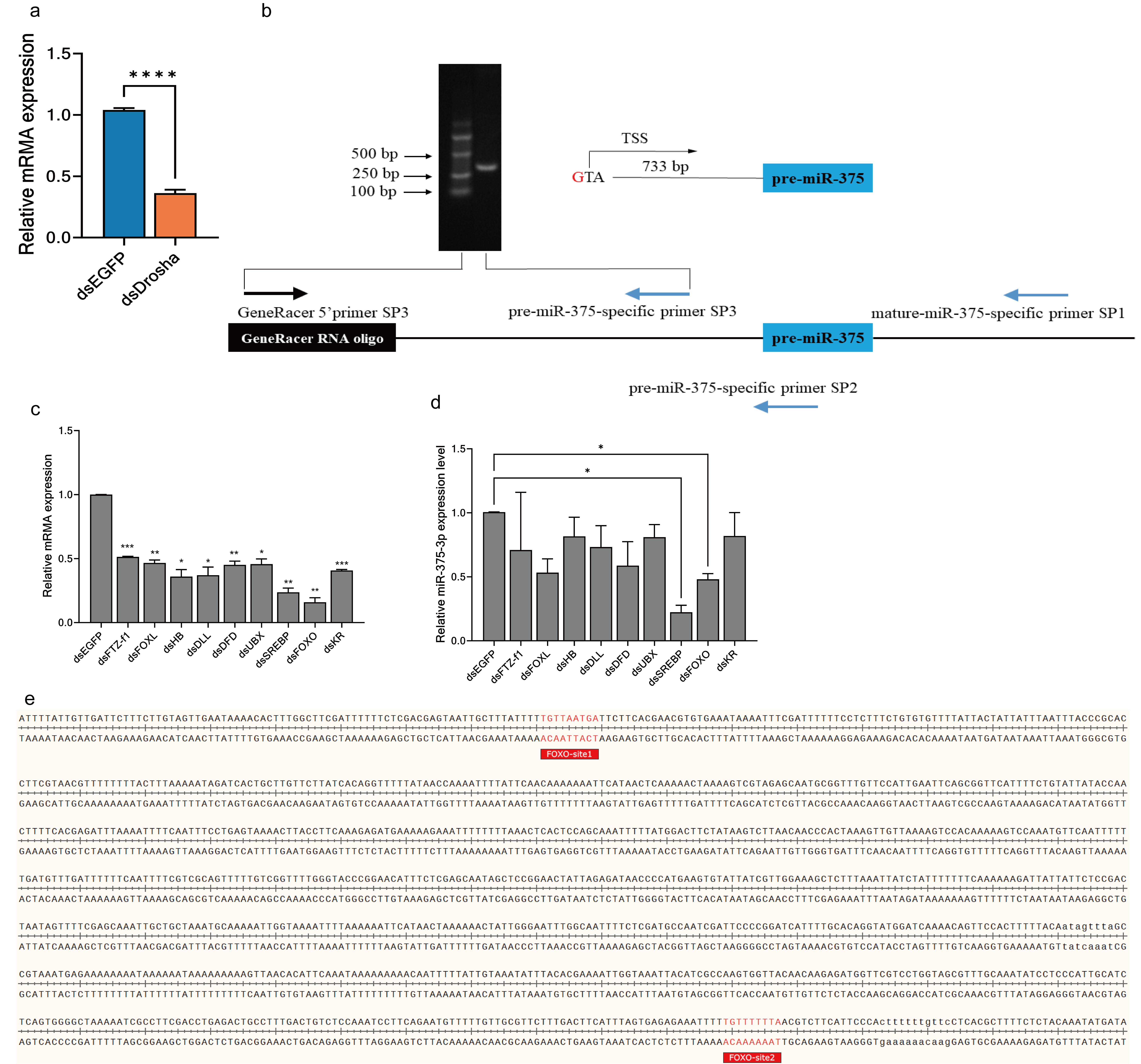


Fig. S8 Screening and identification of transcription factors

a Expression levels of *Drosha* in the beetles based on qPCR. b 5′ RACE shows the transcription start site (TSS). SP1, SP2, and SP3 represent three designed RACE primers, and the PCR bands indicate the length from 1000 bp upstream of the promoter to SP3. c Knockdown efficiency of the predicted transcription factors. d Expression levels of miR-375-3p following transcription factor knockdown. e A schematic diagram of the two predicted binding sites within the promoter region.

Fig. S9


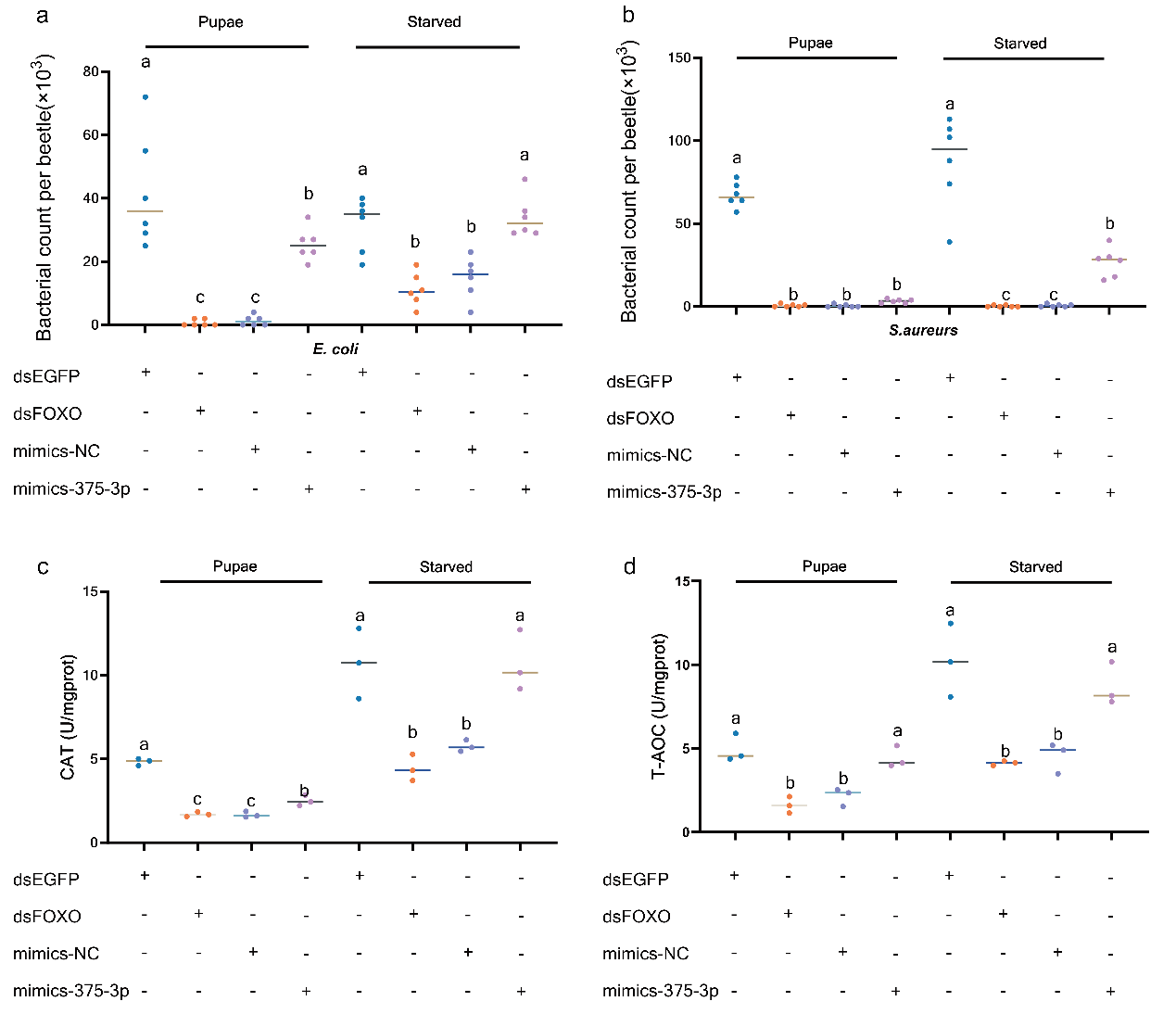


Fig. S9 Foxo regulates immune responses and oxidative stress via miR-375-3p

a The bacterial count of the beetles infected with *E. coli* during pupal, feeding and starvation stage.b The bacterial count of the beetles infected with *S. aureurs* during pupal, feeding and starvation s stage. c Activities of CAT during pupal and starvation stage. d Activities of T-AOC during pupal and starvation stage. (one-way ANOVA followed with Tukey’s test, different letters indicate significant differences within treatment).

Fig. S10


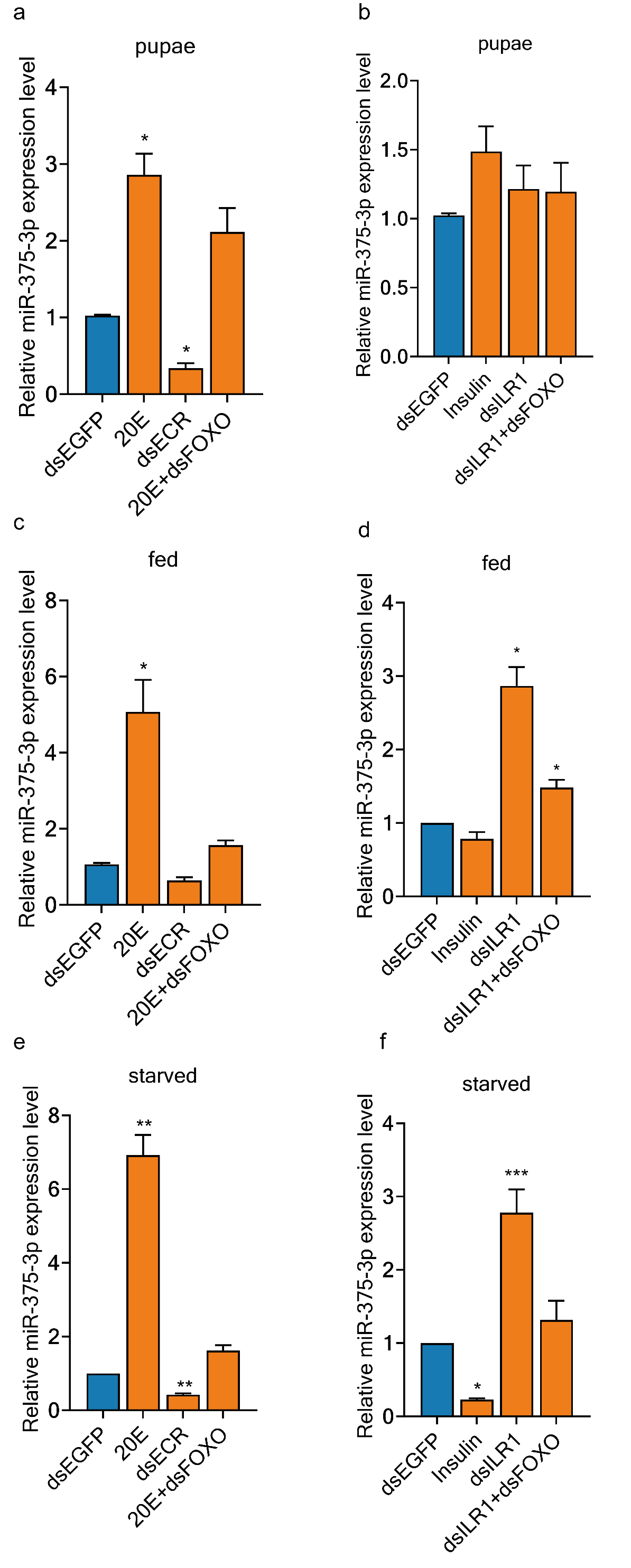


Fig. S10. Comparative analysis of miR-375-3p transcript abundance in activation and inhibition of 20E and insulin pathway.

a-b The expression level of miR-375-3p in larvae under feeding treatment. c-d The expression level of miR-375-3p in larvae under starvation treatment. e-f The expression level of miR-375-3p in larvae under starvation treatment. (Error bars represent SEM. one-way ANOVA followed with Tukey’s test, ***p < 0.001, **p < 0.01 *p < 0.05).

Fig. S11


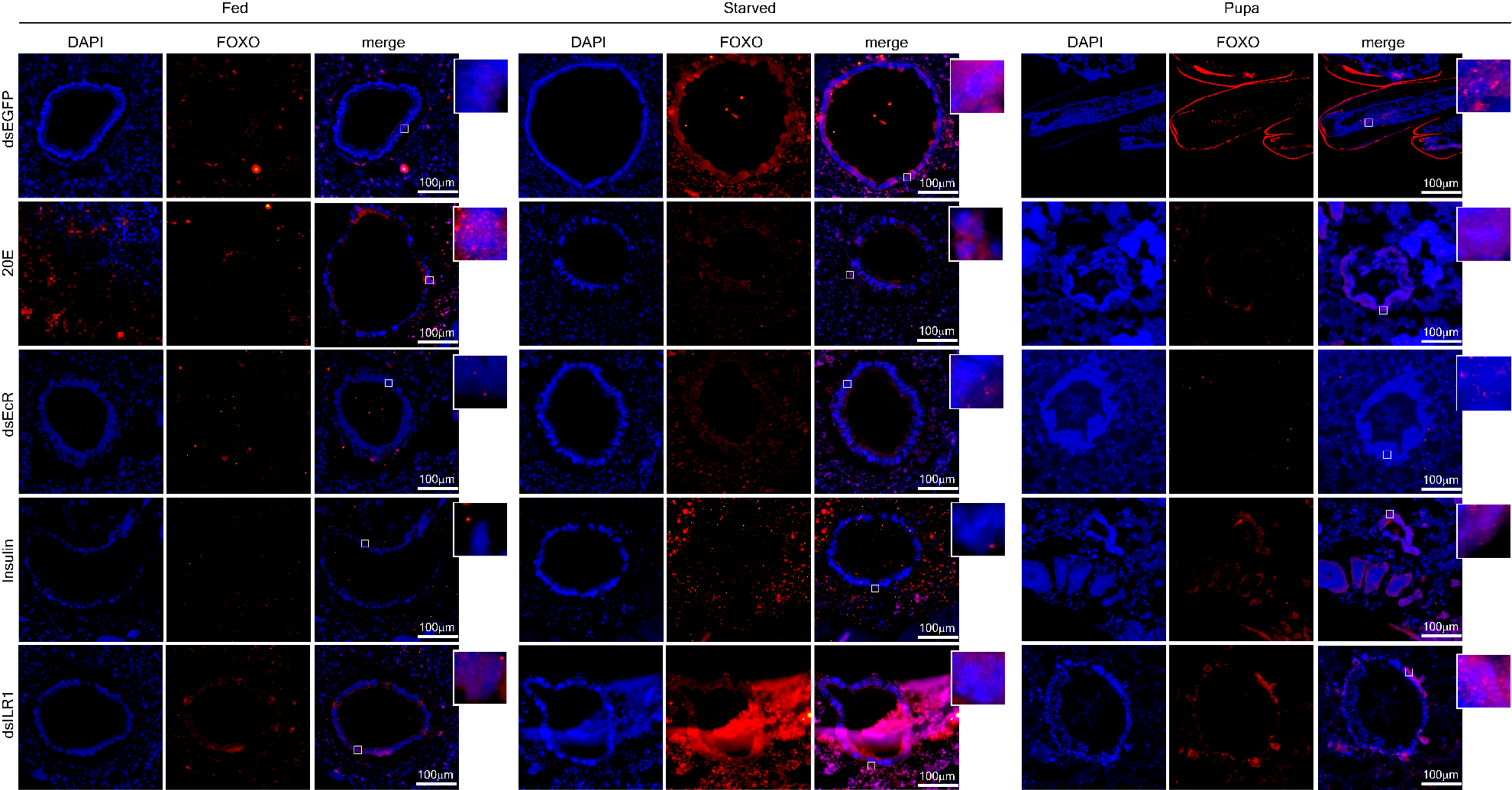


Fig. S11 The nuclear localization of FOXO following various hormone treatments

Blue representing DAPI-stained nuclei and red indicating Cy3-labeled FOXO. The treatments were applied during feeding, starvation, and pupal stages. The experimental groups included *dsEGFP*, 20E injection, insulin injection, and *dsILR1* treatments.

Fig. S12


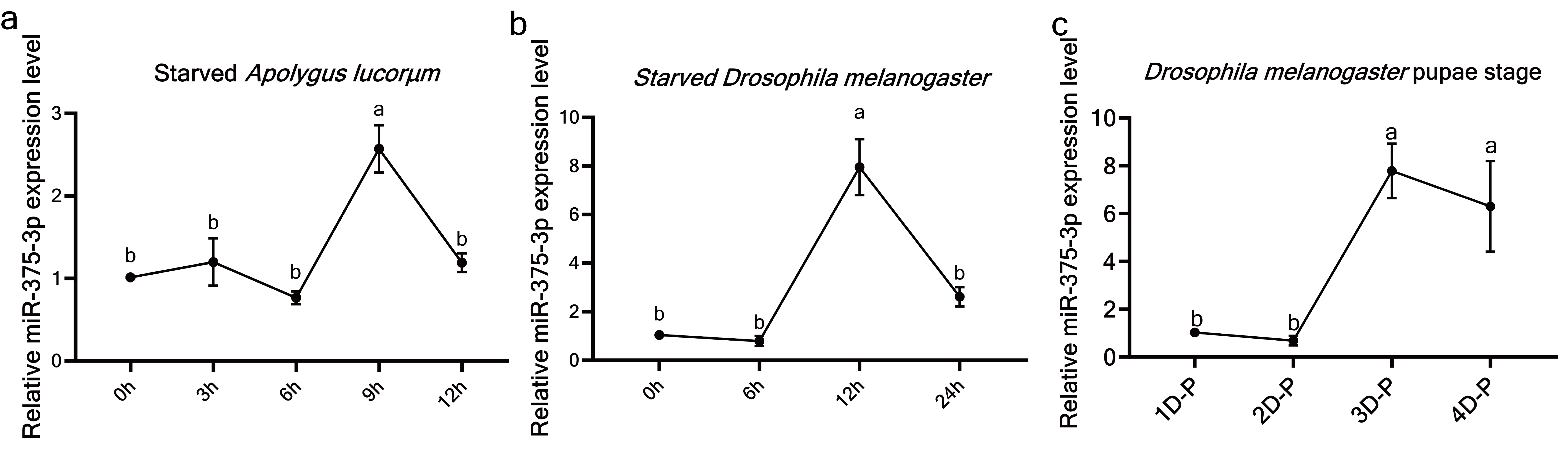


Fig. S12

Expression of miR-375 in *A. lucorum* and *D. melanogaster* after treatment. a The expression level of miR-375-3p in adults under starvation treatment in *A. lucorum.*

b The expression level of miR-375-3p in adults under starvation treatment in *D. melanogaster.* c The expression level of miR-375-3p from pupal stage to adult stage in *D. melanogaster.* (Error bars represent SEM. one-way ANOVA followed with Tukey’s test, P < 0.05 for a and b)
